# Programmed Autophagy in the Fat Body of *Aedes aegypti* Is Required to Maintain Egg Maturation Cycles

**DOI:** 10.1371/journal.pone.0025502

**Published:** 2011-11-17

**Authors:** Bart Bryant, Alexander S. Raikhel

**Affiliations:** Department of Entomology, Institute for Integrative Genome Biology, University of California Riverside, Riverside, California, United States of America; St. Georges University of London, United Kingdom

## Abstract

Autophagy plays a pivotal role by allowing cells to recycle cellular components under conditions of stress, starvation, development and cancer. In this work, we have demonstrated that programmed autophagy in the mosquito fat body plays a critical role in maintaining of developmental switches required for normal progression of gonadotrophic cycles. Mosquitoes must feed on vertebrate blood for their egg development, with each gonadotrophic cycle being tightly coupled to a separate blood meal. As a consequence, some mosquito species are vectors of pathogens that cause devastating diseases in humans and domestic animals, most importantly malaria and Dengue fever. Hence, deciphering mechanisms to control egg developmental cycles is of paramount importance for devising novel approaches for mosquito control. Central to egg development is vitellogenesis, the production of yolk protein precursors in the fat body, the tissue analogous to a vertebrate liver, and their subsequent specific accumulation in developing oocytes. During each egg developmental cycle, the fat body undergoes a developmental program that includes previtellogenic build-up of biosynthetic machinery, intense production of yolk protein precursors, and termination of vitellogenesis. The importance of autophagy for termination of vitellogenesis was confirmed by RNA interference (RNAi) depletions of several autophagic genes (*ATGs*), which inhibited autophagy and resulted in untimely hyper activation of TOR and prolonged production of the major yolk protein precursor, vitellogenin (Vg). RNAi depletion of the ecdysone receptor (*EcR*) demonstrated its activating role of autophagy. Depletion of the autophagic genes and of *EcR* led to inhibition of the competence factor, *betaFTZ-F1*, which is required for ecdysone-mediated developmental transitions. Moreover, autophagy-incompetent female mosquitoes were unable to complete the second reproductive cycle and exhibited retardation and abnormalities in egg maturation. Thus, our study has revealed a novel function of programmed autophagy in maintaining egg maturation cycles in mosquitoes.

## Introduction

Autophagy is highly conserved among metazoans, where bulk degradation of cytoplasmic components is coordinated by means of a lysosomal-mediated pathway via double membrane vesicles. It plays a pivotal role by allowing cells to recycle cellular components under conditions of stress and starvation and developmental transitions [1], [2], [3]. Involvement of autophagy in carcinogenesis has greatly stimulated research of this essential cellular process [4]. The genes responsible for autophagy were first characterized in the yeast *Saccharomyces cerevisiae* [5], [6], [7] and are termed ‘*ATG*’ followed by a number. Orthologues for most of these genes have been found in multiple organisms, with a high level of conservation of functionality across taxa (reviewed in [1]). The steps of autophagy induction and the *ATG* genes that regulate them include: (i) the induction of a double membrane vesicle (*TOR*, *ATG1*, *and ATG 13*), (ii) the nucleation step of the vesicle (*ATG6*, *Vps34*, and -*15*), (iii) vesicle expansion (*ATG3*, -*4*, -*5*, -*7*, -*8*, -*10*, -*12*, and -*16*) and, finally, (iv) recycling of the vesicle (*ATG2*, -*9*, and -*18*) [1], [5], [6], [7], [8].

Programmed autophagy is an integral part of developmental processes, such as dauer formation in nematodes and metamorphosis in fruit flies [3], [9], [10], [11], [12], [13], [14]. During *Drosophila* metamorphosis, larval tissues (midgut, salivary gland, and fat body) undergo autophagic degradation, with *ATG* genes being crucial for this process [10], [11], [12], [13], [14] . Autophagy is negatively regulated by the Target-of-Rapamycin (TOR) signaling pathway, but is induced by *EcR* through regulation of the PI3K pathway in *Drosophila* fat body during late larval development [2], [8], [10], [15].

Mosquito female reproductive biology is unique because egg development is cyclic, and each cycle is linked to intake of vertebrate blood. Consequently, successive gonadotrophic cycles serve as a foundation for transmission of human disease pathogen. Therefore, deciphering the complex biology linking blood feeding and development of eggs for these disease vectors is vital for developing innovative vector control strategies. In the yellow fever mosquito *Aedes aegypti*, used in this study, the female obtains a blood meal and undergoes a process termed vitellogenesis, during which the fat body (a tissue analogous to the mammalian liver and adipose tissue) produces massive amounts of yolk protein precursors (YPPs). These precursors are secreted into the hemolymph and accumulated by developing oocytes via receptor-mediated endocytosis [16], [17]. Ingestion of blood by the female mosquito causes the release of the neurohormone ovarian ecdysiotropic hormone (OEH) and insulin-like peptides from neurosecretory cells in the brain, which, in turn, stimulate ovaries to produce the pre-hormone steroid hormone ecdysone, which is transformed to active 20-hydroxyecdysone (20E) in target tissues [18], [19], [20]. The cooperative action of the nutritional amino acid/TOR signaling and the 20E pathways is responsible for initiation and maintenance of egg development and vitellogenesis in blood-fed, competent female mosquitoes [21], [22], [23].

The mosquito fat body undergoes dramatic changes during the first maturation cycle according to the demands of a reproducing female mosquito, switching from a tissue supporting the energy requirements of a host-seeking insect to one producing massive amounts of YPPs needed for rapid egg development (vitellogenesis), and then back to being a center of energy resources and metabolism [24]. Dramatic decline in *Vg* gene expression and production of its protein at the termination phase of vitellogenesis coincides with the cessation of YPP uptake by developing oocytes and elevation of lysosomal activity in the fat body [25]. As assessed by electron microscopy, at this stage the fat body cells are filled with autophagosomes—cellular organelles surrounded by double membranes—which is a sign of active autophagy [25], [26]. These studies have suggested that autophagy may be involved in the termination of vitellogenesis; however, the biological significance of this process for egg maturation was not clear.

In this work, using molecular biological tools, we have demonstrated that programmed autophagy in the mosquito fat body plays a pivotal role in maintaining of developmental switches required for normal progression of gonadotrophic cycles.

## Results

### Autophagy exhibited up-regulation during the termination phase of vitellogenesis

**Figure 1:**
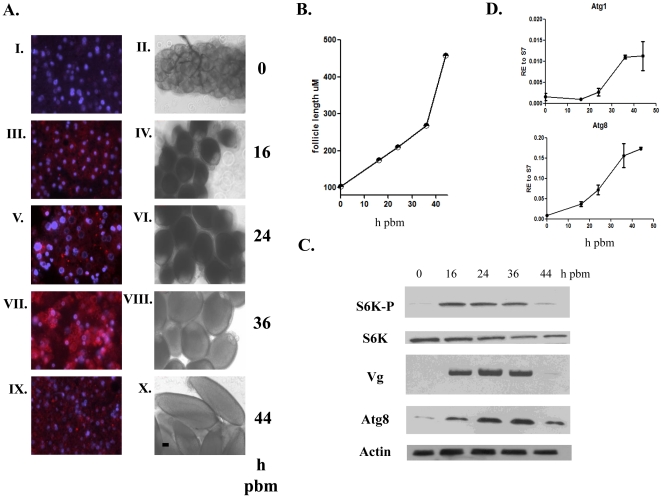
Autophagy is active during the termination phase of vitellogenesis in *A. aegypti* female mosquitoes. (**A**) Autophagy in the fat body of the mosquito was assessed by lysotracker staining at time points 0, 16, 24, 36 and 44 h PBM (A- I, III, V, VII, and IX). Eggs from same batches of mosquitoes are shown. Scale bar (black bar) is 50 µm. (A- II, IV, VI, VIII, and X) where (**B**) follicle length was plotted versus time PBM. Data shown are mean of 5 individual follicles. (**C**) Western blot analyses of utilizing antibodies against phosphorylated S6K (S6K-P), native S6K, Vg, Atg8 and actin. Fat bodies were analyzed at time points 0, 16, 24, 36, and 44 h PBM. (**D**) Transcript analysis of *ATG1* and *ATG8* in female fat bodies at time points 0, 16, 24, 36, and 44 h PBM. Data shown in (D) are three biological replicates and are illustrated as mean ±SEM.

In order to visualize autophagic activity in the mosquito fat body during the first egg maturation cycle, we employed the lysosome-specific fluorescent dye LysoTracker Red, a marker widely used in studies of autophagy [27]. The fat body of previtellogenic females was completely void of lysotracker staining, but bright punctate staining appeared by 16 h post blood meal (PBM). This staining reached maximal intensity at 36 hr PBM, at the time of termination of vitellogenesis and then declined by 44 hr PBM (Fig. 1A). Fig. 1A also shows developing egg chambers (follicles), corresponding to fat bodies analyzed by Lysotracker staining; at 24 h PBM they were ∼211 µM in length and increased to ∼458 µM by 44 h PBM (Fig. 1B). Expression of the *Vg* gene, used as readout for the status of vitellogenesis, peaked at 24 h PBM and then declined (Fig. S1). Transcript levels of most *ATGs*, analyzed by means of quantitative real-time PCR (qPCR), exhibited a similar general trend in fat bodies from blood-fed female mosquitoes, being maximal at 36 h PBM when *Vg* transcript declined (Fig. S1). For further analyses, we selected *ATG1*, *ATG6* and *ATG8*; *ATG1* is a critical initiator of autophagy, *ATG6* is involved in the nucleation step of the vesicle, and *ATG8* in vesicle expansion [1], [5], [6], [7], [8]. *ATG1* and *ATG8* transcripts showed a similar pattern of expression, starting to rise at 12–24 h PBM and reaching their peaks at about 36 h PBM (Fig. 1D). The expression pattern of *ATG6* transcript was different; it was not up-regulated until 36 h PBM (Fig. S1).

**Figure 2:**
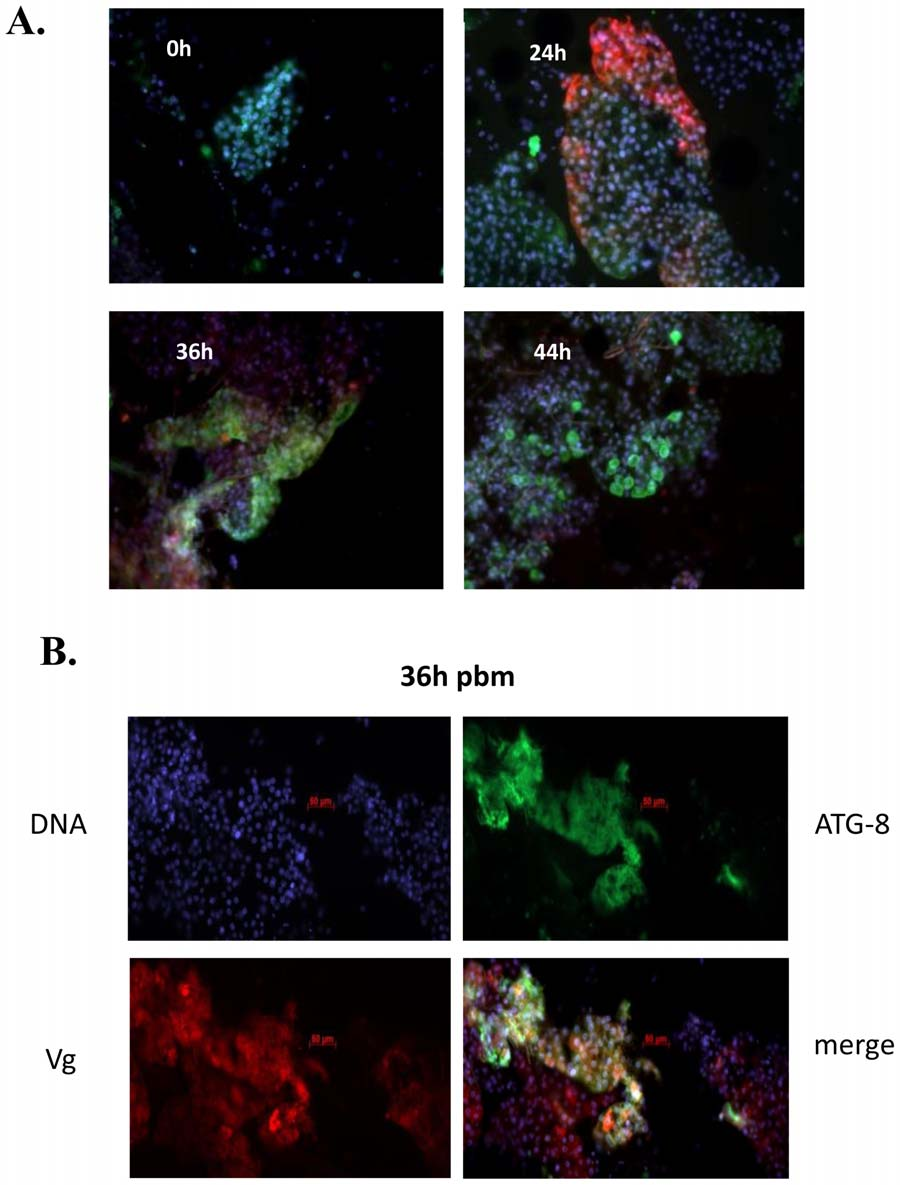
Immunolocalization of Vg and ATG8 within the mosquito fat body. (**A**) Vg and ATG8 were localized by means of fluorescent immunocytochemistry in the fat body at 0, 24, 36, and 44 h PBM. Vg was labeled by monoclonal antibodies against Aedes Vg small subunit (apoprotein), followed by anti-mouse antibodies conjugated to Texas-Red (red), while ATG8 with polyclonal antibodies against Aedes ATG8 followed by anti-rabbit antibodies conjugated to FITC (green). Images were merged. Individual images can be found in Fig. S2. (**B**) Co-localization of ATG8 and Vg in the fat body at 36 h PBM, where ATG8 and Vg were labeled as in (**A**). Labeling is shown separately for ATG8 and Vg and merged, where co-localization is shown as yellow. Nuclei are stained with Hoescht 33342.

To augment characterization of fat body autophagy, we analyzed the localization of the autophagy marker ATG8 in relation to Vg within fat bodies over the course of vitellogenesis using immunofluorescence analysis. Vg was visualized using Vg monoclonal antibodies followed by anti-mouse Texas-Red conjugated antibodies, while ATG8 was labeled with polyclonal antibodies followed by anti-rabbit FITC conjugated antibodies. Immunodetection of ATG8 in fat bodies showed its low basal level at 0 and 24 h PBM with the highest label intensity at 36 h PBM, which decreased by 44 h PBM (Figs. 2A and S2). Vg was undetectable in the same tissue prior to blood feeding (0 h) and, correlating with our western blot analysis, the Vg label exhibited the highest intensity at 24 h PBM (Fig. 2A). By 36 h, the Vg immunofluorescence level was not as intense and at 44 h PBM Vg was again undetectable (Figs. 2A and S2). To control for secondary antibody specificity, we repeated the experiment except that we used ant-mouse secondary antibodies conjugated with FITC for detection of Vg, and anti-rabbit Texas-Red-conjugated antibodies to visualize ATG8. Regardless of the secondary antibody label, we found the same trends as described for the prior experiment (Fig. S3). These immunofluorescence data correlated with above analyses utilizing lysotracker staining, ATG transcript screening, and ATG8 western blot analysis.

While expression of Vg in the fat body at 36 h PBM was overall considerably lower than that at 24 h PBM, we observed many instances of co-localization of Vg and ATG8 at 36 h PBM (Figs. 2B and S3B), suggestive that autophagy could be involved in degradation of Vg in the fat body during the termination phase of vitellogenesis at 36 h PBM (Fig. 2B). This observation was in agreement with a previous electron microscope study showing the presence of vesicles, positively labeled with anti-Vg antibodies, enveloped by double autophagic membranes in the mosquito fat body at 36 h PBM [25], [26]. Only a small population of cells had a high level of the ATG8 autophagy marker, suggesting an increased turnover of some fat body cells after the completion of a vitellogenic cycle (Figs. 2, S2 and S3).

Taken together, these data show that autophagy is an integral process of the programmed termination of vitellogenesis in the fat bodies of blood-fed reproducing female mosquitoes.

### Termination of vitellogenesis was delayed in autophagy-incompetent mosquitoes

**Figure 3:**
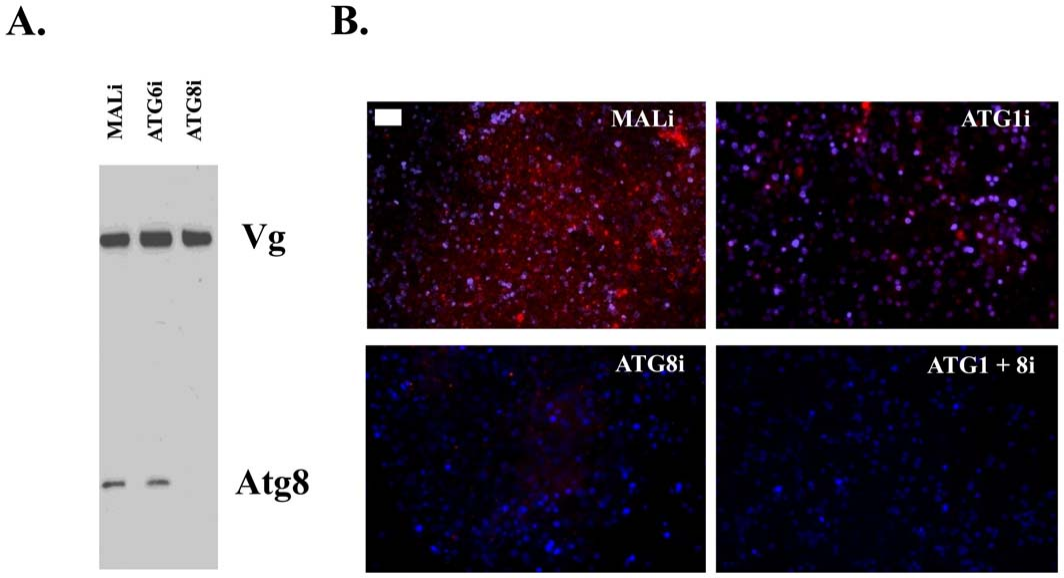
*ATG1* and *ATG8* are required for autophagy induction in the *A. aegypti* female fat body. (**A**) ATG8i knockdown specificity was tested using immunoblot analysis. ATG8 RNAi resulted in a depletion of ATG8 protein; while it was present after either Mali or ATG6i treatments. The level of Vg was not affected after any treatment. ATG8 was detected by anti-Drosophila ATG8 antibody and Vg by monoclonal antibodies against Aedes Vg. Fat bodies from female mosquitoes 24 h PBM. (**B**) Both *ATG1* and *ATG8* were knocked down in single and double RNAi experiments, where these backgrounds were assessed for lysotracker staining of the fat body at 36 h PBM. Note stronger effect of the double *ATG1*+*ATG8* RNAi depletion than individual RNAi of either this ATGs. Mali did not exhibit any changes in lysotracker staining compared to untreated control (Fig. 1A, VII). Scale bar (white bar) is 50 µm.

To decipher the role of autophagy in the programmed progression of vitellogenesis in the fat bodies of reproducing female mosquitoes, we employed reverse genetics and performed experiments with RNAi depletion of *ATG1* and *ATG8*. We injected 1- to 2-day-old female mosquitoes with the following combinations of dsRNA molecules: *ATG1*, *ATG8*, or *ATG1+ATG8*. *MAL* was used as a negative control, as described previously [28], and application of dsMal did not affect progression of vitellogenesis (Figs. S5 and S6), which was similar to that in untreated female mosquitoes (Figs. 1C, S2 and S3). In all RNAi experiments, RNA levels of these autophagic genes were significantly diminished, showing their efficient depletion at the transcript level (Fig. S4). In addition, immunoblot analysis showed that ATG8 was sufficiently depleted at the protein level (Fig. 3A). We analyzed the effectiveness of RNAi depletion of *ATG1* and *ATG8* in fat bodies at the peak of autophagic activity at 36 h PBM by means of lysotracker staining. MALi control exhibited a high level of autophagic activity, as judged by the intensity of lysotracker staining (Fig. 3B). In contrast, RNAi depletion of *ATG1* and *ATG8* resulted in a significant reduction of lysotracker staining in fat bodies (Fig. 3B). Fat bodies with depleted *ATG1* contained low punctate lysotracker staining, and the effect of *ATG8* depletion was stronger. Significantly, a double depletion of *ATG1* and *ATG8* illustrated the lowest lysotracker staining (Fig. 3B). These data indicate the importance of *ATG1* and *ATG8* genes for the induction of autophagy in the fat body of blood-fed female mosquitoes at the time of termination of vitellogenesis.

**Figure 4:**
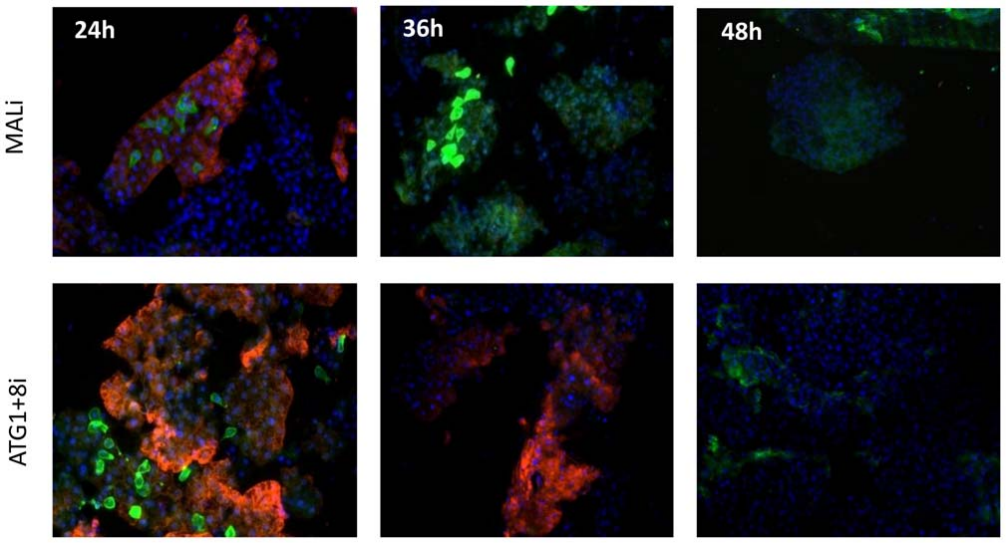
Autophagy-incompetent mosquitoes are unable to terminate vitellogenesis in a timely manner. ATG8 and Vg expression was assessed by means of immunofluorescence in the fat body at 24, 36 and 48 h PBM in MALi and ATG1+8i backgrounds. ATG8 is labeled with polyclonal antibodies against Aedes ATG8 followed by secondary anti-rabbit antibodies conjugated to FITC (green) and Vg with monoclonal antibodies against Aedes Vg small subunit followed by secondary anti-mouse antibodies conjugated to Texas-RED (red). Only merged images are illustrated. Individual images can be found in Figs S6 and S9.

**Figure 5:**
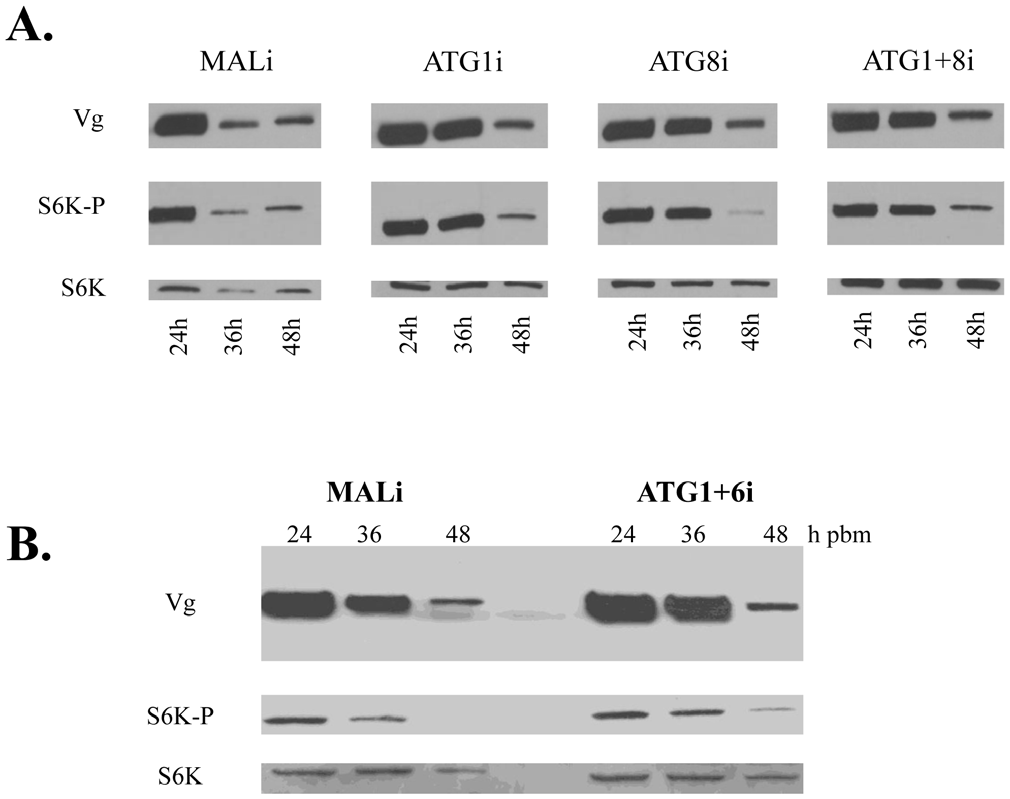
RNA interference depletions of several autophagic genes (*ATGs*) resulted in untimely activation of TOR and prolonged production of Vg. (**A, B**) Western blot analyses utilizing antibodies against phosphorylated S6K (S6K-P), Vg and native S6K. (A) S6K-P phosphorylation and Vg production in fat bodies from MALi, ATG1i, ATG8i, and ATG1+8i (A) and ATG1+6i (B) backgrounds at 24, 36 and 48 h PBM. Native S6K was used as a loading control.

Next, we asked whether the inhibition of autophagy would affect the progression of vitellogenesis in blood-fed females. We visualized Vg and ATG8 within the fat bodies of these autophagy-incompetent mosquitoes at 24, 36 and 48 h PBM by means of immunofluorescence. In these experiments, Vg was labeled with Vg monoclonal antibodies followed by anti-mouse Texas-Red conjugated antibodies, while ATG8 with ATG8 polyclonal antibodies followed by anti-rabbit FITC conjugated antibodies. At 36 h PBM, ATG8 was no longer present in the ATG8i and ATG1+8i backgrounds, unlike MALi control, which had a high level of ATG8 (Fig. 4 and Figs. S6, S7, S8, S9). In contrast to normal progression of vitellogenesis as signified by MALi control, which had very low levels of Vg in fat bodies at 36 h PBM, Vg was abundantly present in fat bodies in all of the autophagy-incompetent mosquitoes at this time point (Fig. 4 and Fig. S6, S7, S8, S9). We found that the highest prevalence of Vg was in the double knock-down background ATG1+8i, when compared with the other backgrounds, which is consistent with a stronger inhibition of autophagy in mosquitoes with this double depletion (Fig. 4 and Fig. S9). Western blot analysis confirmed that Vg levels were elevated in fat bodies of these autophagy-incompetent mosquitoes at 36 h PBM (Fig. 5A). To obtain additional confirmation of these results, we also tested another autophagy-incompetent background (ATG1+6i). Both *ATG1* and *ATG6* were efficiently knocked down after *ATG1*+*ATG6*i depletion (Fig. S4), and *ATG1*+*ATG6*i depletion completely eliminated lysotracker staining in fat bodies at 36 h PBM (Fig. S10). Western blot analysis revealed that fat bodies from these autophagy-incompetent mosquitoes had an elevated Vg level at 36 h PBM (Fig. 5B). These results suggested the importance of autophagy in the programmed termination of vitellogenesis.

### Autophagy-incompetent mosquitoes exhibited a delay in termination of TOR activity

Nutritional signaling mediated by the TOR pathway plays a pivotal role in initiation of vitellogenesis in mosquitoes [21]. TOR activity monitored by the phosphorylation status of S6K is high after a blood meal and decreases thereafter, becoming undetectable by the time of termination of vitellogenesis [22]. To establish a background for monitoring TOR activity, we used the same approach, which showed that the phosphorylation status of S6K peaked early in vitellogenesis (Fig. 1C). In the same fat body samples, Vg protein was maximally elevated at 24 h PBM and ATG8 at 36 h PBM, demonstrating sequential overlapping activities in this tissue: TOR activation, production of YPPs, and autophagy (Fig. 1C). As previously shown for Drosophila, ATG1 is a negative regulator of TOR, resulting in down regulation of S6K phosphorylation [2], [15], [29]. Therefore, we asked whether TOR activity was affected in fat bodies of autophagy-incompetent mosquitoes. We analyzed the phosphorylation status of S6K in fat bodies of autophagy-deficient mosquitoes at 36 and 48 h PBM, when S6K activity is normally low. We found an elevated phosphorylation level of S6K in all tested autophagy-incompetent backgrounds: *ATG1*i, *ATG8*i, and *ATG1*+*ATG8*i (Fig. 5A). Analysis of mosquitoes with another autophagy-incompetent background (*ATG1*+*ATG6*i) also showed an elevated phosphorylation level of S6K in their fat bodies at 36 h PBM (Fig. 5B). Immunoblot screening of the same fat body samples in Figs. 5A and 5B revealed that these elevated levels of S6K phosphorylation correlated with increased levels of Vg.

Collectively, these data show that autophagy is a negative regulator of TOR signaling and Vg production in the fat body of female mosquitoes during the termination phase of vitellogenesis. Failure of timely activation of autophagy leads to elevated TOR activity and prolonged vitellogenesis.

### *EcR* was involved in induction of autophagy in mosquito vitellogenic fat bodies

**Figure 6:**
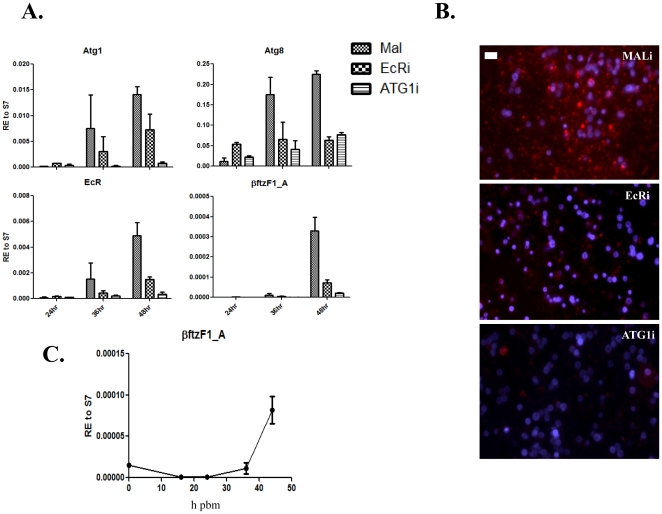
Effect of *EcR* and *ATG1* depletions. (**A**) *EcR* was depleted by RNAi and compared with ATG1i (positive control) and MALi (negative control) for transcript analysis of *ATG1*, *ATG8*, *EcR*, and *betaFTZ-F1_A* at 24, 36, and 48 h PBM in fat bodies of female mosquitoes. Data shown are two or three biological replicates and presented as mean ±SEM. Data are normalized relative to S7. (**B**) Fat bodies from *EcR*i, *ATG1*i, and MALi were analyzed for lysotracker staining at 36 h PBM. Scale bar (white bar) is 20 µm. (C) Fat bodies from untreated female mosquitoes were analyzed for expression of *betaFTZ-F1_A* at time points 0, 16, 24, 36, and 44 h PBM.

In mosquitoes, 20E is a key regulator of egg development and vitellogenesis, and the action of 20E is mediated by the EcR, which is a heterodimer consisting of EcR and USP [30], [31]. We wanted to know whether 20E is involved in the regulation of developmental progression of fat body activities by controlling not only the activation of vitellogenesis but also its termination. To determine whether *EcR* plays any role in autophagy induction in the fat body of the female mosquito during egg development, we investigated the occurrence of this event following *EcR* RNAi depletion experiments. We used *ATG1*i as a positive control and MALi as negative control. Transcripts of both *ATG1* and *EcR* were efficiently depleted by their respective RNAi treatments (Fig. 6A), and fat bodies of both backgrounds were negative for lysotracker staining at 36 h PBM when compared with MALi (Fig. 6B). In both *ATG1*i and *EcR*i backgrounds, induction of the *ATG8* transcript was hindered, relative to that in the MALi control, which showed up regulation of *ATG8* at 36 and 48 h PBM (Fig. 6A). Induction of *ATG1* was also inhibited in the *EcR*i background (Fig. 6A). Taken together, these data suggest that *EcR* is involved in induction of autophagy at the termination phase of vitellogenesis. In the *ATG1*i background, *EcR* also failed to be properly up-regulated at 36 and 48 h PBM (Fig. 6A).

### Mosquitoes with disrupted autophagy were unable to up regulate the competence factor *betaFTZ-F1*

**Figure 7:**
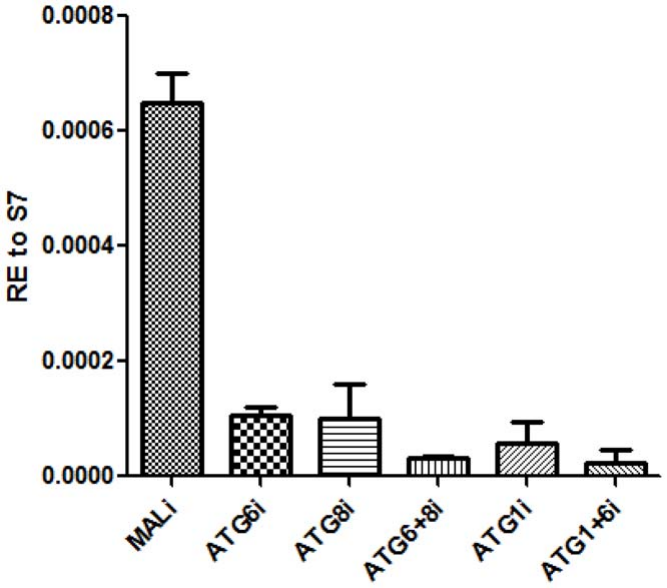
Autophagy-incompetent mosquitoes failed to induce competence factor *betaFTZ-F1_A*. Autophagy-incompetent backgrounds (ATG1i, ATG6i, ATG8i, ATG6+8i, and ATG1+6i) were analyzed for up-regulation of the competence factor *betaFTZ-F1_A* at 48 h PBM. Data shown are two or three biological replicates and are illustrated as mean ±SEM. An unpaired Student's *t* test was used for comparison for all ATG-incompetent backgrounds compared with MALi. All comparisons to MALi had significant *P* values of <0.05.

The nuclear receptor betaFTZ-F1—called the competence factor—is important for maintaining 20E-regulated developmental switches during development and metamorphosis of *Drosophila melanogaster* [32]. In *Ae. aegypti*, this nuclear receptor is required for the onset of vitellogenesis and successful egg development in the first cycle, during which it is highly expressed in the pre-vitellogenic stage and then declines during the synthesis stage of vitellogenesis [33]. In addition, *betaFtz-F1* is expressed again at the cessation of vitellogenesis following up regulation of *HR3*, presumably in preparation for the next vitellogenic cycle [33]. More recently, it has been shown that there are two betaFTZ-F1 isoforms in *A. aegypti* [34]. In our experiments, *betaFtz-F1_A* was up-regulated in the fat body during the late termination period, 48 h PBM, after sequential activation and inhibition of *Vg* gene expression and subsequent activation of autophagic genes (Fig. 6C). RNAi depletion of *EcR* prevented *betaFtz-F1_A* induction in the fat body at 48 h PBM in accordance with the proposed role of the ecdysone hierarchy in regulation of *betaFtz-F1* (Fig. 6A). Surprisingly, induction of *betaFtz-F1_A* was also inhibited in the ATG1i background (Fig. 6A). Because of this unexpected finding, we tested different autophagy-incompetent backgrounds for *βFtz-F1_A* expression in fat bodies at 48 h PBM. We examined expression of this nuclear receptor in either single-knockdown or double-knockdown experiments: *ATG1*i, *ATG6*i, *ATG8*i, *ATG6*+*ATG8*i, and *ATG1*+*ATG6*i. All *ATG* genes were efficiently knocked down in all backgrounds tested (Fig. S4) and, in congruence with the *EcR*i and *ATG1*i backgrounds, *betaFtz-F1_A* failed to be properly induced at 48 h PBM in any of the autophagy-incompetent backgrounds (Fig. 7).

### The second cycle of egg development was obstructed in autophagy-incompetent mosquitoes

**Figure 8:**
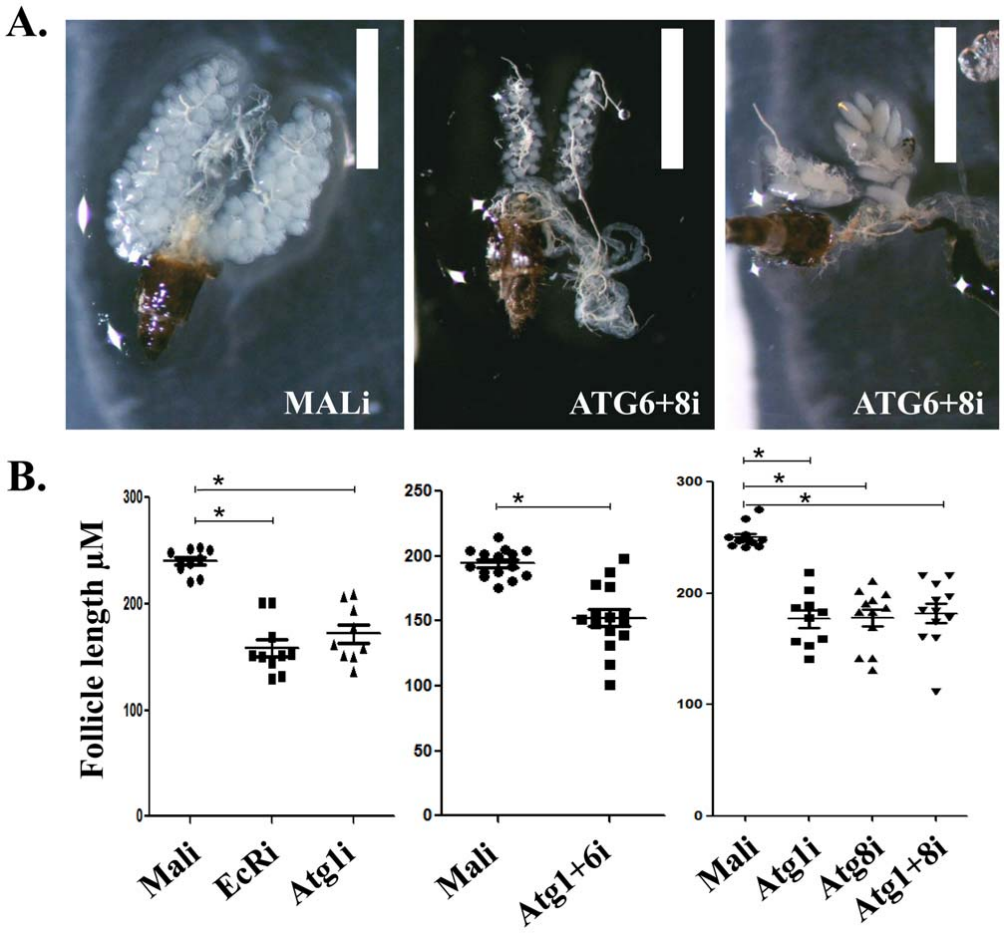
Second cycle of egg development was severely affected in autophagy-incompetent mosquitoes. (**A**) Autophagy-incompetent background ATG6+8i demonstrated severe defects in egg development, illustrating two phenotypes: (i) small or (ii) fewer in number and bigger, compared with the negative control MALi. Scale bar is 1 mm. (**B**) Follicle lengths for multiple autophagy-incompetent backgrounds (EcRi, ATG1i, ATG1+6i, ATG8i, and ATG1+8i) were smaller than for MALi control. Data shown are individual follicle sizes and are illustrated as mean ±SEM. For EcRi and ATG1i (*n* = 10). For ATG1+6i (*n* = 15). For ATG1i, ATG8i, ATG1+8i (*n* = 12). An unpaired Student's *t* test was used for comparison. *P* values<0.0001 are designated with *.

Owing to this dramatic disruption of fat body functions in autophagy-incompetent mosquitoes, we tested their ability to mature eggs. For these experiments, we examined autophagy-incompetent mosquitoes with several combinations of RNAi depletions of *ATG* genes. These mosquitoes exhibited no disruption of blood feeding and developed normal first batches of eggs. 3–4 days after oviposition of eggs, these autophagy-incompetent mosquitoes were blood fed again and examined at 24 h PBM of the second gonadotrophic cycle. We found that autophagy-incompetent mosquitoes were unable to properly develop a second batch of eggs when compared with MALi. Ovaries from mosquitoes with *ATG6*+*ATG8*i depletion are shown as typical representatives of these phenotypes (Figure 8A). As in *ATG6*+*ATG8*i, in all the other autophagy-incompetent backgrounds tested (*ATG1*i, *ATG1+ATG6i*, *ATG8*i, and *ATG1+ATG8i*), smaller follicle length and more variance in follicle length were illustrated compared to negative control MALi (Fig. 8B). In all cases, ovarian development was severely compromised (Fig. 8A and 8B). Most ovaries remained small, but in some autophagy-incompetent backgrounds, we observed ovaries simultaneously containing follicles that varied from 100 (resting previtellogenic stage) to 400 µM in length (Fig. 8A). In contrast, mosquitoes with the MALi background had ovaries with uniformly sized follicles 200–250 µM in length, which corresponds to normally developing follicles at 24 h PBM (Figs. 8A, 8B and Fig. 1B). These data illustrated the importance of programmed fat body autophagy in the female mosquito for maintaining cyclicity of blood-meal-dependent egg development.

RNAi depletion of *EcR* in the first gonadotrophic cycle disrupted programmed fat body autophagy (Fig. 6). Mosquitoes with RNAi-depleted *EcR* background also failed to develop ovaries in the second gonadotrophic cycle (Fig. 8B). The ovarian phenotype of this *EcR* depletion was similar to those of autophagy-incompetent mosquitoes. Although *EcR* depletion likely affects multiple targets in a reproducing female mosquito, the role of EcR in activating fat body autophagy provides further evidence of the importance of this process for normal progression of gonadotrophic cycles.

## Discussion

### Programmed autophagy is required for a timely termination of vitellogenesis

Although molecular mechanisms of autophagy are remarkably conserved among eukaryotic organisms, this process plays various roles essential for their growth, development and survival under stress conditions, [1], [2], [3]. For many developmental events, autophagy is implicated in terminal degradation of tissues. During metamorphosis of *Drosophila*, larval organs and tissues, the salivary glands, the midgut and the fat body, are degraded to permit morphogenesis of adult tissues [3], [10], [11], [12], [13], [14]. In contrast, we report here a case of programmed autophagy that is involved in developmental remodeling of the female mosquito fat body, which occurs without degradation of this tissue. We show that highly regulated autophagy is required for aiding functional reprogramming of the fat body during egg developmental cycles in the mosquito. This programmed autophagy is required for a timely termination of vitellogenesis in the female mosquito fat body as illustrated by means of RNAi depletions of *ATG* genes.

A tight link between TOR signaling and autophagy has been demonstrated in *Drosophila* larvae during starvation [1], [2], [29]. Overexpression of *ATG1* in the fat body of these larvae is sufficient to lower the TOR activity and induce high levels of autophagy [8]. In contrast, in *ATG1* ‘loss-of-function’ mutant flies, TOR is hyperactive as determined by S6K phosphorylation status [15]. Monitoring of S6K phosphorylation levels showed that TOR activity sharply increased in the fat body of the female mosquito after it obtained a blood meal, peaking at 16 h PBM and decreasing thereafter in a trend opposite to the autophagy markers. This suggested an inverse relationship for these two pathways. Indeed, RNAi depletion of *ATG1* alone or in a combination with either *ATG8* or *ATG6* resulted in an elevated and prolonged TOR activation through 48 h PBM as could be judged from elevated phosphorylation level of S6K. Thus, it appears that the negative feedback loop between TOR and autophagy is conserved in the fat body of vitellogenic female mosquitoes. Whether the programmed autophagy acts on termination of vitellogenesis in the mosquito fat body via its inhibition of TOR remains to be elucidated.

### EcR is involved in induction of autophagy in mosquito vitellogenic fat bodies

20E, the main hormone orchestrating metamorphic developmental changes in holometabolous insects, has been implicated in activating autophagy [35], [36], [37], [38]. Studies utilizing *Drosophila* have clearly shown a critical role for 20E signaling in the activation of programmed developmental autophagy [3], [10], [11], [12]. Ectopic overexpression of *EcR* in the *Drosophila* fat body induces autophagy, while fat body expression of a dominant-negative *EcR* in the mid third instar larvae resulted in a reduction of autophagy [10]. *Drosophila* genetic studies further suggest that 20E/EcR acts by counteracting an inhibitory function of the PI3K pathway and activating the nuclear receptor E93 [3], [10], [11], [12]. In this study, we show that *EcR* is an activator of autophagy in the mosquito fat body during termination of vitellogenesis. Similar to insect metamorphosis, 20E is the major regulator of mosquito vitellogenesis and controls *YPP* gene expression in the fat body [23], [24]. A precise timing of *Vg* gene expression is regulated by alternative action of 20E hierarchy factors; synergistic action of EcRB/USPB, E74B, and Br Z2 activates a high level of *Vg* gene expression, while Br isoforms Z1 and Z4 are involved in a timely termination of this gene expression [31], [39], [40], [41], [42]. In this current study, we have shown that expression of *ATG1* and *ATG8* genes was inhibited after EcR RNAi depletion, strongly suggesting that 20E/EcR is involved in regulation of *ATG* genes. This is likely accomplished by a unique combination of 20E hierarchy factor isoforms, determination of which is an important goal for the future research. Taken together, these data indicate that 20E is the master regulator of the developmental program determining the precise timing of events during mosquito egg maturation cycles.

### Autophagy is essential for developmental transitions during egg maturation cycles

The competence factor betaFTZ-F1 is required for 20E-mediated developmental switches during insect development, metamorphosis, and reproduction [32], [42], [43]. In this study, we have identified a regulatory link between autophagy and betaFTZ-F1 during termination of the first vitellogenic cycle in the mosquito fat body. RNAi depletions of *ATG* genes inhibited elevation in *betaFtz-F1_A* expression at 36 and 48 h PBM; this was confirmed by testing RNAi knockdowns of several *ATG* genes individually and in combination: *ATG1*, *ATG6*, *ATG8*, *ATG6+ATG8*, and *ATG1+ATG6*. The precise mechanism of autophagy involvement in activating *betaFtz-F1* in the remodeling mosquito fat body is unclear at this time. The role of 20E hierarchy in regulation of *betaFtz-F1* activation is well established for the metamorphic developmental switch in Drosophila [32], [43], [44], [45]. In the late third instar larva, a high titer of 20E mediated by EcR/USP activates the early genes *E74A*, *E75A*, and *Br*, products of which are responsible for activation of target genes and underlying biological responses. At the same time, EcR/USP activates late expression of the nuclear receptor *HR3*, which in turn inhibits *E74A*, *E75A*, and *Br*, and activates *betaFtz-F1*. Activation of *betaFtz-F1* requires a low titer of 20E and occurs in mid-prepupa. *betaFtz-F1* provides competence for the early genes to be reactivated by 20E in the late prepupal stage. In the mosquito, overlapping expression of *HR3* and *betaFtz-F1* has been observed during egg maturation cycles: *HR3* is expressed during late pupal–early adult development and is followed by the elevation in *betaFtz-F1*; then the *HR3* transcript is enhanced again at 36 h PBM, followed by another rise in *betaFtz-F1* [33], [34]. *In vitro* fat body culture experiments have shown that *HR3* is activated by 20E, while *betaFtz*-F1 is inhibited; this is in accordance with observed *in vivo* fluctuating expression patterns of these nuclear receptors during egg maturation cycles [34]. A reverse genetics approach has clearly demonstrated the requirement of *betaFtz-F1* for the mosquito fat body competence to 20E response and further studies have revealed the molecular basis of *betaFtz-F1* action as a competence factor in which it recruits the p160/SRC coactivator, FISC, to EcR/USP [40], [42]. RNAi depletion of *ATG1* inhibited post-vitellogenic induction of both *EcR* and *betaFtz-F1* in the mosquito fat body. Hence, it is likely that *ATG1* plays an activating role in the late 20E regulatory circuit, elevating *EcR* expression, which in turn triggers induction of *betaFtz-F1*. The late activation of *betaFtz-F1* in the mosquito fat body was also prevented by RNAi depletion of HR3 (Daniel Mane-Padros and Alexander Raikhel, unpublished data), suggesting that HR3 is likely involved in this late 20E regulatory circuit However, because of technical limitation of the RNAi approach in mosquitoes, in which dsRNA is introduced into previtellogenic female mosquitoes, we cannot rule out that EcR RNAi depletion did not have an effect on earlier events regulated by the 20E hierarchy, such as activation of E74, E75 and Br after blood feeding. In turn, repression of these factors would lead to mis-regulation of the downstream targets including *EcR* itself and *betaFtz-F1*. These possible effects should be investigated in the future.

Additional confirmation of the requirement of autophagy for the developmental transition during egg maturation cycles was provided by our data that the second cycle of egg development was obstructed in autophagy-incompetent mosquitoes. Depletion of *ATG* genes was performed in mosquitoes prior to the first blood meal and a subsequent first egg developmental cycle. These mosquitoes fed on blood and produced a normal first batch of eggs, showing that depletion of *ATG* genes did not affect the egg maturation despite its effect on the fat body. The second blood feeding of these mosquitoes was not affected, although they were unable to properly develop a second batch of eggs. Hence, disruption of autophagic remodeling during the termination of the first cycle affects the normal progression of the developmental transition to the second egg maturation cycle. The fat body is an essential metabolic tissue, and its remodeling is clearly required to support the reproducing female mosquito. In addition, as has been shown for the Drosophila fat body, it carries a signaling role, producing a factor that activates the brain to secrete insulin-like peptides (ILPs) [46]. *A. aegypti* ILPs have metabolic and gonadotrophic regulatory functions, with ILP-3 being involved in elevating carbohydrate and lipid storage in sugar-fed adult females [18], [19], [20], [47]. Whether, programmed autophagy is required to maintain the fat body's signaling role in the context of cyclic egg production is an important question to answer in the future. *EcR* RNAi depletion also rendered mosquitoes incapable of developing a second batch of eggs. This observation indicates the regulatory connection between autophagy and the 20E pathway, as discussed above. Lastly, because of systemic nature of RNAi approach in mosquitoes, we cannot completely exclude a possibility of an additional effect of depletion of *ATG* genes on development of secondary ovarian follicles in mosquito ovaries, which start maturing at the end of the first egg developmental cycle. Recent reports in *Drosophila* have shown that autophagy is required for progression of oogenesis [48], [49].

In conclusion, in this study we characterized the role of autophagy in reproducing female mosquitoes. We have clearly shown that autophagy is essential for maintaining developmental transitions between egg maturation cycles. Female mosquitoes acquire and transmit disease pathogens during successive blood feedings, which support egg developmental cycles. Interrupting transition to a subsequent reproductive cycle has obvious detrimental effects on female fecundity and would lead to a decrease in mosquito population and a reduction of pathogen transmission. A large body of information has been obtained concerning regulation of the first egg developmental cycle in mosquitoes [23], [24]. However, this is the first report to decipher the regulatory circuitry governing developmental switches between the cycles. Our study has demonstrated the importance of understanding how this important disease vector insect regulates its cyclic egg production.

## Materials and Methods

### Animals

The mosquito *A. aegypti* UGAL/Rockefeller strain was raised as described previously [28]. Female mosquitoes 3–5 days post-eclosion were fed on the blood of anesthetized white rats to initiate egg development. All procedures for using vertebrate animals were approved by the University of California Riverside Institutional Animal Care and Use Committee (#A20100016; 05/27/2010).

### RNA expression analysis

RNA was isolated by Trizol (Invitrogen) extractions from fat bodies of blood-fed female mosquitoes at various time points. RNA was digested with DNAse I (cat # 18068015 Invitrogen), and then DNA-digested RNA was subjected to cDNA synthesis with SuperScript II (cat # 18064-014). cDNA was used as a template for expression analysis with SYBR green (cat # 170-8882 Bio-Rad) using the following PCR conditions: Step 1 = 95°C for 3 min. Step 2 = 95°C for 30 s, 55°C for 30 s and 72°C for 30 s; this step was repeated 50 times. Step 3 = 95°C for 1 min. This was followed by melt curve analysis. Quantitative real time PCR (qPCR) was done on an iCycler iQ (Bio-Rad). Primers for expression analysis are found in Table S1. Expression was plotted using 2^−ΔCt^, where the cycle threshold (Ct) for the gene of interest is compared with the Ct of the housekeeping gene *S7* [50]. *S7* primers are S7-Forward primer 5′ TCAGTGTACAAGAAGCTGACCGGA 3′ and S7-Reverse primer 5′ TTCCGCGCGCGCTCACTTATTAGATT 3′.

### RNA interference

Gene models for the mosquito *ATG* genes have been reported previously [51] and can be found at http://cegg.unige.ch/Insecta/immunodb. For RNAi experiments, PCR products were cloned into the TOPO-TA vector from Invitrogen (cat# 45-0640) following the manufacturer's specific instructions. For *EcR*, primers were designed to the common region of EcR, yielding a 289-bp region for dsRNA production. Primers for this construct were EcR-RNAi_forward 5′ AACAACCGGTCCTACACGAG 3′ and EcR-RNAi_reverse 5′ TCAGGATCGACAGCAGTTTG 3′. For *ATG1*, primers were designed to yield a 350-bp region used for dsRNA production. Primers for this construct were ATG1-RNAi_forward 5′ CCTGACTGGTAAGGCACCAT 3′ and ATG1-RNAi_reverse 5′ GTTGTTGCTGCTGGAGTTGA 3′. For *ATG6*, primers were designed to yield a 351-bp region for dsRNA production. Primers for this construct were ATG6-RNAi_forward 5′ GCACCGAAGGGACGTTATTA 3′ and ATG6-RNAi_reverse 5′ CCATACAACGGCAGTTCCTT 3′. For *ATG8*, primers were designed to yield a 252-bp region for dsRNA production. Primers for this construct were ATG8-RNAi_forward 5′ GGAAGAACACCCATTCGAGA 3′ and ATG8-RNAi_reverse 5′ GTAGCCGATGTTGGTGGAAT 3′. Once the plasmid constructs were verified by sequencing, they were used to make dsRNA. To obtain an amplicon for use as a substrate for a dsRNA reaction, the following primers were used: Univ. TOPO forward primer 5′ taatacgactcactatagggGATCCACTAGTAACGGCCG 3′ and Univ. TOPO reverse primer 5′ taatacgactcactatagggGTGTGATGGATATCTGCAGAATTCG 3′. Upper case correlates to a common region outside of the PCR product insertion site of the pCR® II vector. Lowercase correlates to the T7 primer sequence. PCR products using the above-mentioned plasmids as templates and the universal primers result in both 5′ and 3′ addition of T7 primer sequence. This PCR product was then used to make dsRNA with the MEGAscript kit from Ambion (cat# AM1334) following the manufacturer's specific instructions.

Female mosquitoes, 1–2 days after eclosion, were CO_2_ anesthetized and injected into the thorax at a volume of 0.5 µl with appropriate dsRNA molecules at 3 µg/µl for both single and double RNAi depletion experiments. *Mal* was used as a negative control, as described previously [21]. Mosquitoes were allowed to recover for 4–5 days before blood feeding.

### Western blot analysis

Protein analysis of Vg and TOR signaling were done according to Hansen et al.[21]. Briefly, fat bodies from blood-fed female mosquitoes were obtained at various time points PBM. Fat bodies were then homogenized in lysis buffer (50 mM Tris HCl, pH 7.4, 1% NP-40, 0.25% sodium deoxycholate, 150 mM NaCl, 1 mM EDTA, 1 mM PMSF, 1× Phosphatase inhibitor from Sigma cat# P2850, and 1× Protease inhibitor from Sigma cat # P8340) and run on Tris-Glycine gels (Invitrogen) before being transferred to PVDF membranes. For detection for Vg protein, a mixture of Vg monoclonal antibodies [52], [53] was used at the 1∶5000 dilution; this was followed by the secondary anti-mouse-HRP (cat # sc-2005 Santa Cruz) at the 1∶2000 dilution. For detection of phosphorylated S6K, the anti-human S6K-P antibody, recognizing a conserved Tyr 388 (Upstate Millipore, cat # 07-018), was used at the 1∶200 dilution followed by the secondary anti-rabbit-HRP (cat # 7074 Cell Signaling) at the 1∶1000 dilution. S6K protein was used as a loading control and, for its detection; we used the polyclonal antibody against human S6K from Santa Cruz (cat # sc-230) at the 1∶100 dilution and the secondary anti-rabbit-HRP at the 1∶1000 dilution, as above. For detection of actin, we used the primary monoclonal antibody against βactin (Sigma) at the 1∶5000 dilution followed by the secondary antibody anti-mouse-HRP at the 1∶3000 dilution. For ATG8 protein analysis, we initially obtained the Drosophila ATG8 antibody [54] (generously provided by Dr. S. Cherry, University of Pennsylvania School of Medicine, Philadelphia, PA). We then produced polyclonal antibodies to *A. aegypti* ATG8 using a commercial vendor, ProSci (http://www.prosci-inc.com/). Polyclonal rabbit anti-ATG8 was generated against the peptide FEKRKAEGDKIRRKYPERVPV and serum was affinity purified by ProSci. For detection of ATG8 protein, both of these antibodies were used at the 1∶500 dilution; this was followed by application of anti-rabbit-HRP secondary antibodies at the 1∶1000 dilution.

### Lysotracker staining, immunofluorescence antibody staining, and oocyte analysis

For lysotracker analysis, fat bodies from female mosquitoes were dissected in Aedes physiological solution (APS) [28] and incubated in APS containing Lysotracker and DNA stains—200 nM of LysoTracker Red DND-99 (L7528 Invitrogen) and 5 µM Hoechst 33342 (H1399 Invitrogen)—for 5–10 min. Fat bodies were then placed on glass slides, covered with glass cover slips and visualized under a Zeiss, AxioObserver A1 microscope.

For immunofluorescence antibody staining, the following procedure was followed. Fat bodies were dissected in APS and fixed in 3.7% formaldehyde (APS as diluent) for 20 min. Fat bodies were then rinsed with APS-T (APS containing 0.3% Triton-X 100) three times for 5 min each. Fat bodies were then incubated in APS-T-BSA (3% BSA, cat # SP-5050 Vector Labs) for 1 h to block possible non-specific binding. The tissue was rinsed again with APS-T, as outlined above, and incubated with primary antibodies diluted in APS-T overnight at 4°C. Fat bodies were then washed, as outlined above, and incubated with secondary antibodies in APS-T for 2 h. The following conjugated secondary antibodies from Vector Labs (http://www.vectorlabs.com/default.aspx) were used in our analyses: anti-rabbit Texas Red (cat # TI-1000), anti-mouse Texas Red (cat # TI-2000), anti-mouse FITC (cat # FI-2000), and anti-rabbit FITC (cat # FI-1000). After incubation with secondary antibodies, fat bodies were washed again, as outlined above, and incubated in APS containing 5 µM Hoechst 33342 to visualize nuclei for 10 min. The processed tissue was then mounted using VectaShield (cat # H-1000, Vector Labs) and analyzed under a Zeiss AxioObserver A1 microscope.

The following dilutions of primary and secondary antibodies were used for immunofluorescence analyses: Vg mouse monoclonal antibodies at 1∶250 with 1∶100 for secondary (either anti-mouse Texas-Red or anti-mouse FITC); ATG8 rabbit polyclonal antibody at 1∶50 with 1∶100 for secondary (either anti-rabbit Texas-Red or anti-rabbit FITC). We initially performed single antibody staining for either ATG8 or Vg to obtain optimal conditions. Once this was accomplished, double antibody staining for ATG8 and Vg was done. In either case—single or double antibody—we found the same trend as outlined in the results. To control for the possibility of false-positive signals from the immunofluorescence analysis, fat bodies from blood-fed female mosquitoes were incubated with secondary antibodies without primary antibody. In all secondary antibody combinations (anti-ms-RED and anti-Rb-FITC; anti-ms-FITC and anti-Rb-RED) and all time point conditions (0, 24, 36, and 44 h PBM), we found low to no background fluorescence when compared with foreground fluorescence. The time point 36 h PBM is shown as a representative (Fig. S11). For accurate comparison analysis, exposure time to excite the fluorophore was always the same between time points and/or different genetic backgrounds. Also both immunofluorescence and lysotracker images were analyzed using AxioVision software.

To measure effects of various treatments on mosquito ovarian development, we measured the length of developing eggs, called follicles or egg chambers. Ovaries were dissected from mosquitoes with various backgrounds in APS. Ovaries and individual follicles were then examined under a Zeiss AxioObserver A1 at the bright field setting. Images were analyzed using AxioVision software.

## Supporting Information

Figure S1 **Temporal expression of** ***ATG*** **gene transcripts during vitellogenesis in the** ***A. aegypti*** **female fat body.** Fat bodies from blood fed mosquitoes at 0, 12, 24 and 36 hr PBM were analyzed for expression of multiple autophagy genes with Vg as the marker for the status of vitellogenesis by means of qPCR. Data shown are two biological replicates and are illustrated as mean ±SEM.
(TIF)

Figure S2 **ATG8 and Vg co-localized within the fat body of female mosquitoes during vitellogenesis.** ATG8 and Vg expression was analyzed by immunofluorescence within the fat body at 0, 24, 36 and 44 h PBM where ATG8 was labeled with polyclonal ATG8 antibody followed by anti-rabbit FITC-conjugated antibodies (green) and Vg was labeled with Vg monoclonal antibodies followed by anti-mouse Texas-RED-conjugated antibodies (red). Stains are shown as individual and merged, where co-localization is shown as yellow.
(TIF)

Figure S3 **Utilization of a different combination of secondary antibodies for localization of ATG8 and Vg within the fat body of female mosquitoes during vitellogenesis.** (**A**) ATG8 and Vg expression was analyzed by immunofluorescence within the fat body at 0, 24, 36 and 44 h PBM where ATG8 was labeled with polyclonal ATG8 antibody followed by Texas-RED- conjugated anti-rabbit antibody (red) and Vg was labeled with Vg monoclonal antibodies followed by anti-mouse FITC antibodies (green). (**B**) Co-localization of ATG8 and Vg at 36 h PBM where ATG8 and Vg were labeled as in (A). Stains are shown as individual and merged, where co-localization is shown as yellow.
(TIF)

Figure S4 **Knockdown efficiency of** ***ATG1*** **, -** ***6*** **, -** ***8*** **in single or double RNAi backgrounds.** To determine knockdown efficiency by RNAi fat bodies from blood fed mosquitoes were analyzed at 36 h PBM, where in all cases the *ATG* gene in question was sufficiently knocked down in both single and double knock down experiments. Data shown are two or three biological replicates and are illustrated as mean ±SEM. An unpaired Student's *t* test was used for comparison and all graphs had significant *P* values<0.05.
(TIF)

Figure S5 **dsMal has no effect on progression of vitellogenesis.** Vg, ATG8 and actin were visualized by their respective antibodies in fat bodies from mosquitoes at 0–44 h PBM.
(TIF)

Figure S6 **Vg and ATG8 immunofluorescence analysis in MALi background.** ATG8 and Vg expression was assessed by immunofluorescence within the fat body at 24, 36 and 48 h PBM in MALi background. ATG8 was labeled with polyclonal ATG8 antibody followed by anti-rabbit FITC-conjugated antibodies (green) and Vg was labeled with Vg monoclonal antibodies followed by anti-mouse Texas-RED-conjugated antibodies (red).
(TIF)

Figure S7 **Vg and ATG8 immunofluorescence analysis in ATG1i background.** ATG8 and Vg expression was assessed by immunofluorescence within the fat body at 24, 36 and 48 h PBM in ATG1i background. ATG8 was labeled with polyclonal ATG8 antibody followed by anti-rabbit FITC-conjugated antibodies (green) and Vg was labeled with Vg monoclonal antibodies followed by anti-mouse Texas-RED-conjugated antibodies (red).
(TIF)

Figure S8 **Vg and ATG8 immunofluorescence analysis in ATG8i background.** ATG8 and Vg expression was assessed by immunofluorescence within the fat body at 24, 36 and 48 h PBM in ATG8i background. ATG8 was labeled with polyclonal ATG8 antibody followed by anti-rabbit FITC-conjugated antibodies (green) and Vg was labeled with Vg monoclonal antibodies followed by anti-mouse Texas-RED-conjugated antibodies (red).
(TIF)

Figure S9 **Vg and ATG8 immunofluorescence analysis in ATG1+8i background.** ATG8 and Vg expression was assessed by immunofluorescence within the fat body at 24, 36 and 48 h PBM in ATG1+8i background. ATG8 was labeled with polyclonal ATG8 antibody followed by anti-rabbit FITC-conjugated antibodies (green) and Vg was labeled with Vg monoclonal antibodies followed by anti-mouse Texas-RED-conjugated antibodies (red).
(TIF)

Figure S10 **Autophagy-incompetent background ATG1+6i are unable to properly induce autophagy.** Fat bodies from MALi or ATG1+6i backgrounds were assessed for lysotracker staining 36 hr PBM. Scale bar of 50 µm is shown in red.
(TIF)

Figure S11 **Control testing of secondary antibody staining of the fat body at 36 hr PBM in the absence of primary antibody.** Fat bodies from blood fed mosquitoes at 36 h PBM were incubated with secondary antibodies without any primary antibody to illustrate the lack of non-specific binding for these antibodies. Scale bar of 50 µm is shown in red.
(TIF)

Table S1 **Primers for expression analysis.**
(TIF)
