# Supplementary figures and images for: Programmed Autophagy in the Fat Body of Aedes aegypti Is Required to Maintain Egg Maturation Cycles
Source: PLoS One. 2011 Nov 17;6(11):e25502. doi: 10.1371/journal.pone.0025502 (PMC3219638; doi:10.1371/journal.pone.0025502)

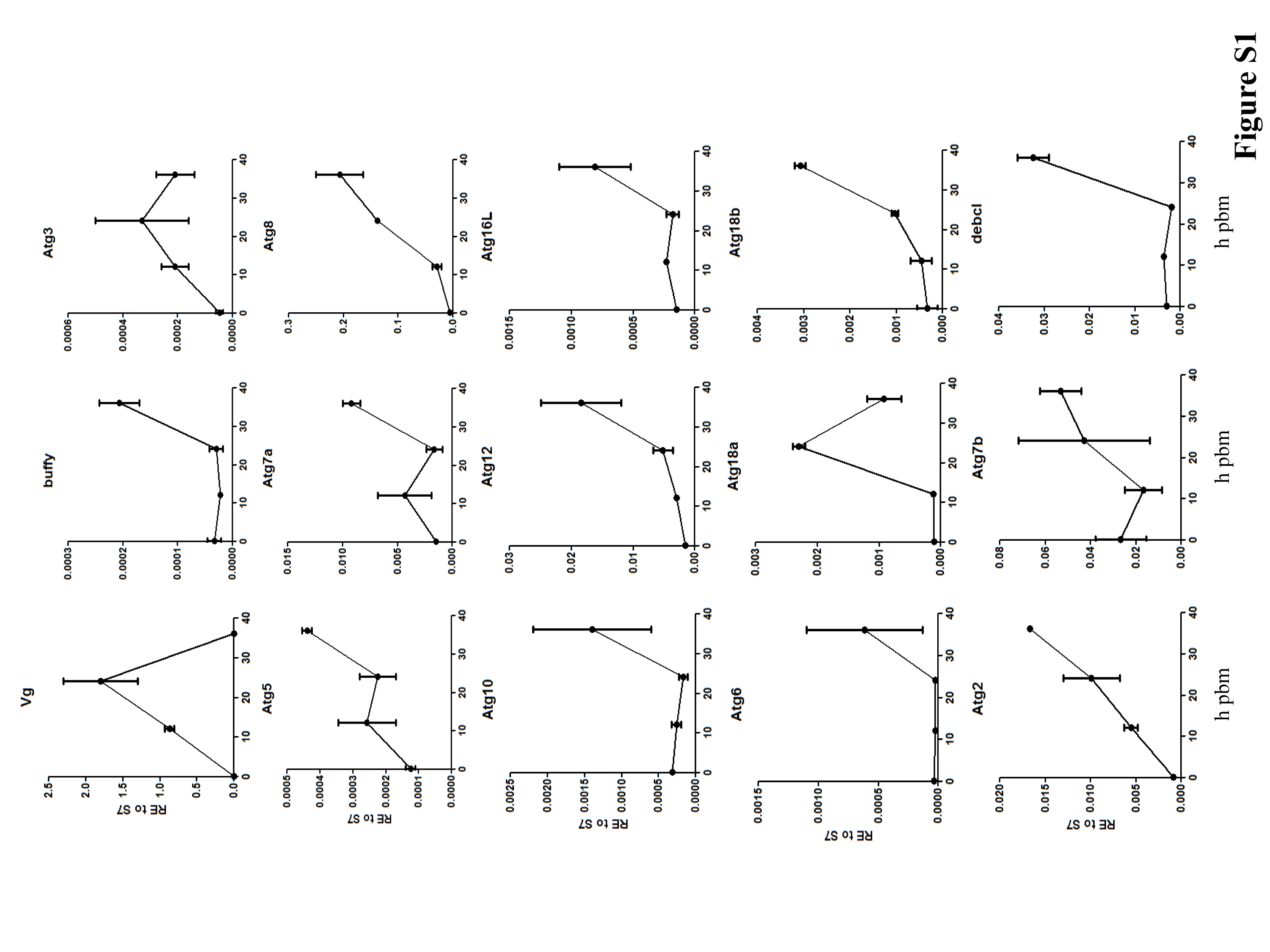

Supplement: Figure S1 — Temporal expression of ATG gene transcripts during vitellogenesis in the A. aegypti female fat body. Fat bodies from blood fed mosquitoes at 0, 12, 24 and 36 hr PBM were analyzed for expression of multiple autophagy genes with Vg as the marker for the status of vitellogenesis by means of qPCR. Data shown are two biological replicates and are illustrated as mean ±SEM. (TIF) [file pone.0025502.s001.tif]

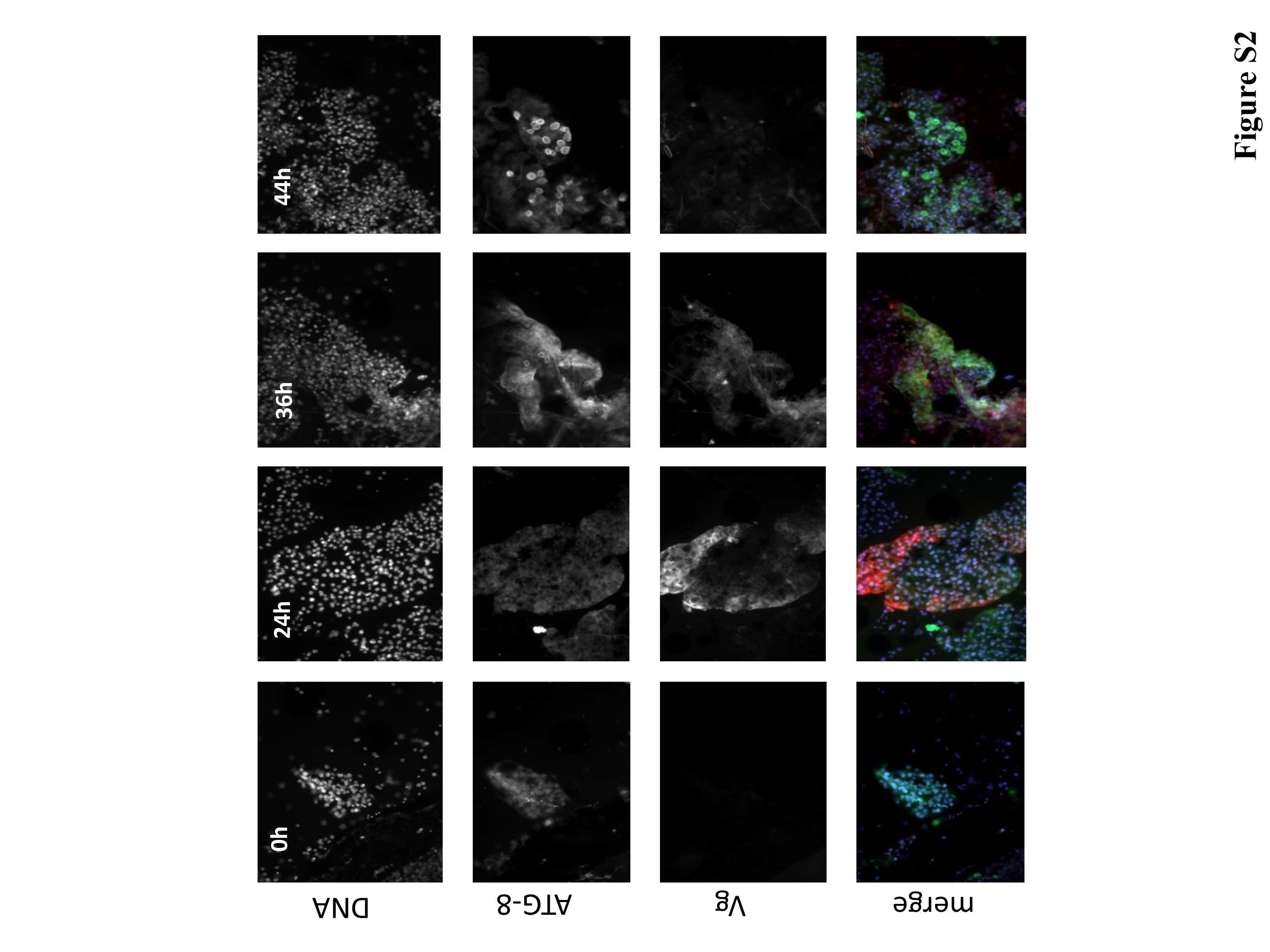

Supplement: Figure S2 — ATG8 and Vg co-localized within the fat body of female mosquitoes during vitellogenesis. ATG8 and Vg expression was analyzed by immunofluorescence within the fat body at 0, 24, 36 and 44 h PBM where ATG8 was labeled with polyclonal ATG8 antibody followed by anti-rabbit FITC-conjugated antibodies (green) and Vg was labeled with Vg monoclonal antibodies followed by anti-mouse Texas-RED-conjugated antibodies (red). Stains are shown as individual and merged, where co-localization is shown as yellow. (TIF) [file pone.0025502.s002.tif]

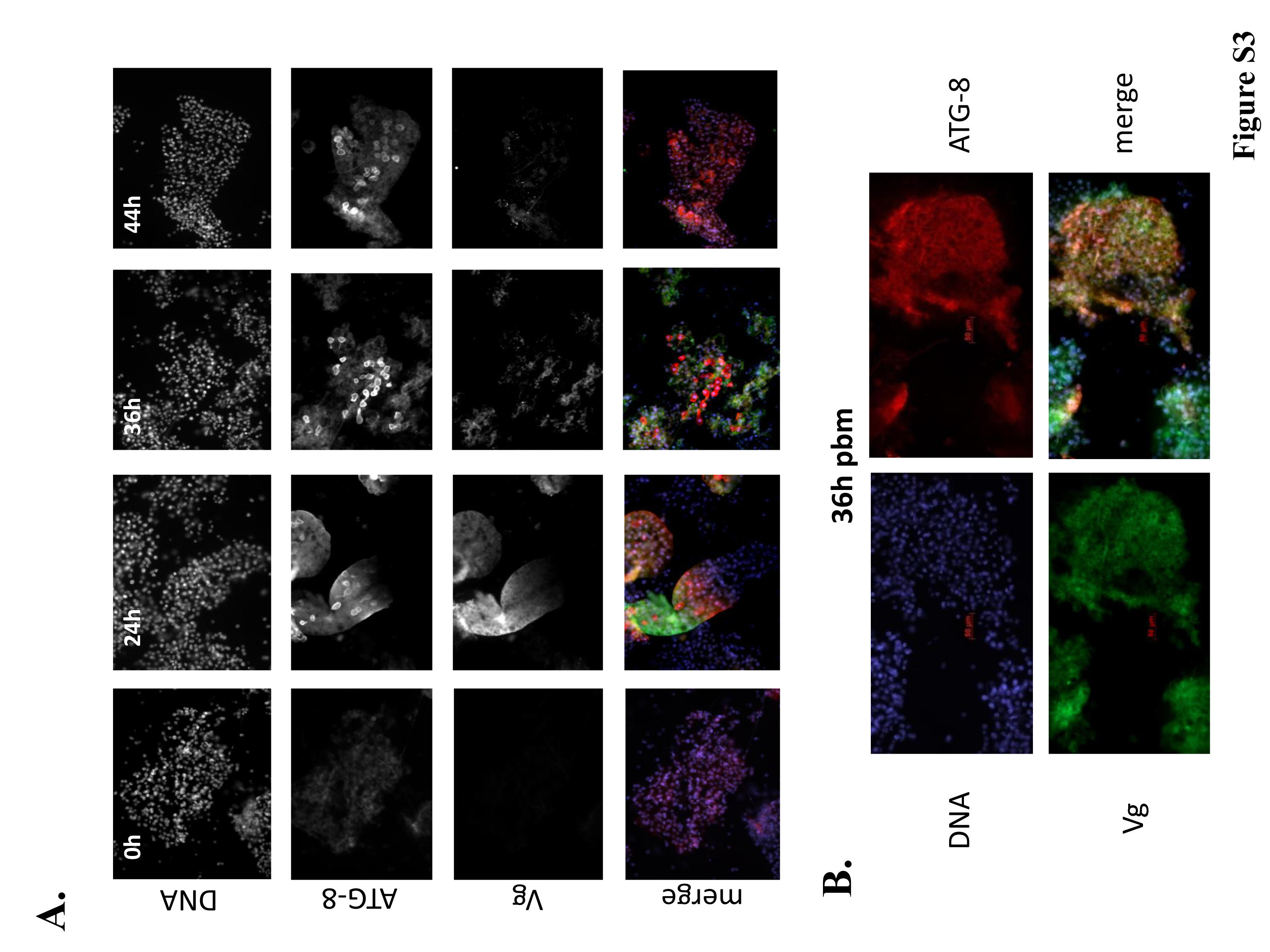

Supplement: Figure S3 — Utilization of a different combination of secondary antibodies for localization of ATG8 and Vg within the fat body of female mosquitoes during vitellogenesis. (A) ATG8 and Vg expression was analyzed by immunofluorescence within the fat body at 0, 24, 36 and 44 h PBM where ATG8 was labeled with polyclonal ATG8 antibody followed by Texas-RED- conjugated anti-rabbit antibody (red) and Vg was labeled with Vg monoclonal antibodies followed by anti-mouse FITC antibodies (green). (B) Co-localization of ATG8 and Vg at 36 h PBM where ATG8 and Vg were labeled as in (A). Stains are shown as individual and merged, where co-localization is shown as yellow. (TIF) [file pone.0025502.s003.tif]

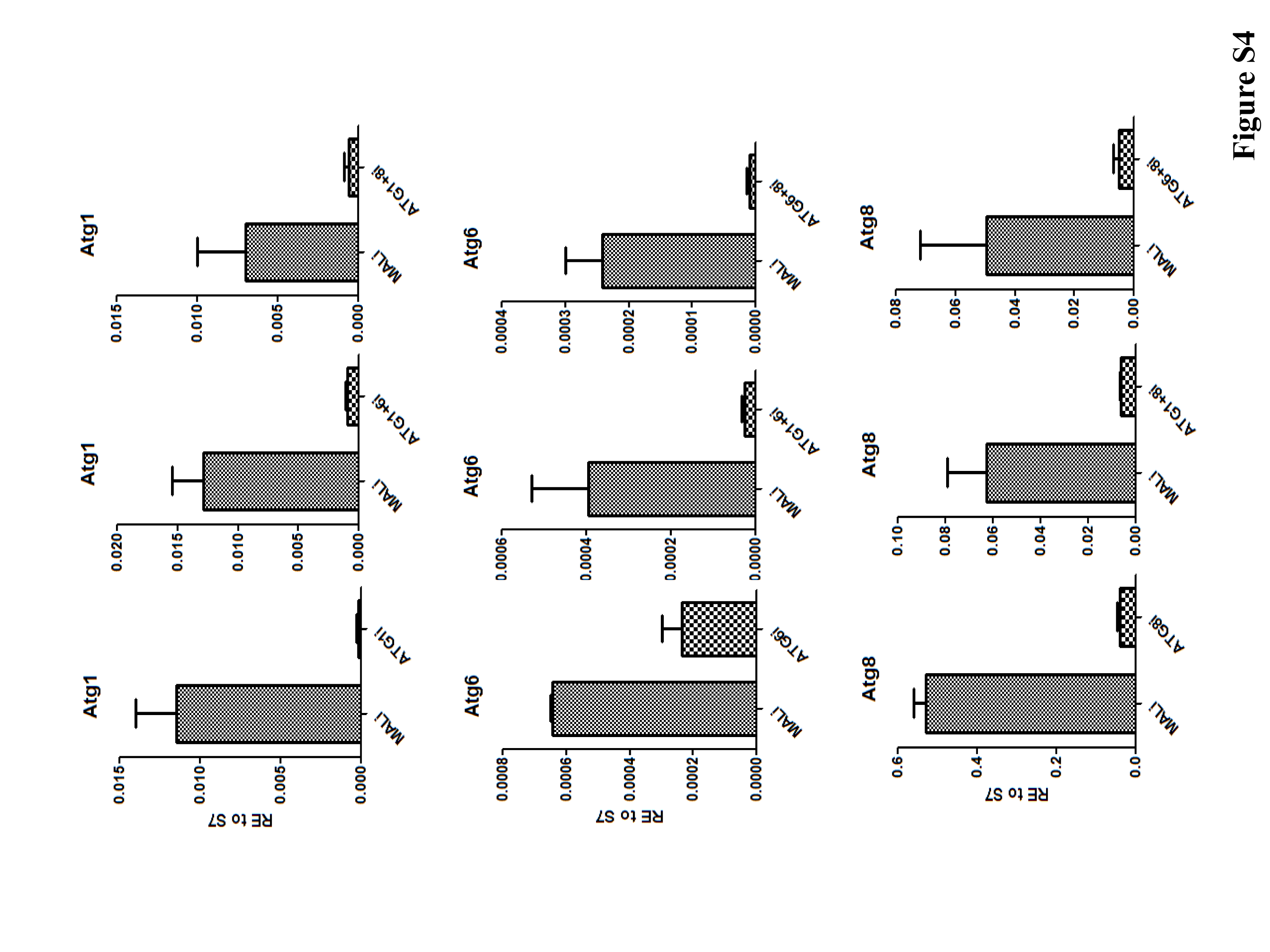

Supplement: Figure S4 — Knockdown efficiency of ATG1 , - 6 , - 8 in single or double RNAi backgrounds. To determine knockdown efficiency by RNAi fat bodies from blood fed mosquitoes were analyzed at 36 h PBM, where in all cases the ATG gene in question was sufficiently knocked down in both single and double knock down experiments. Data shown are two or three biological replicates and are illustrated as mean ±SEM. An unpaired Student's t test was used for comparison and all graphs had significant P values<0.05. (TIF) [file pone.0025502.s004.tif]

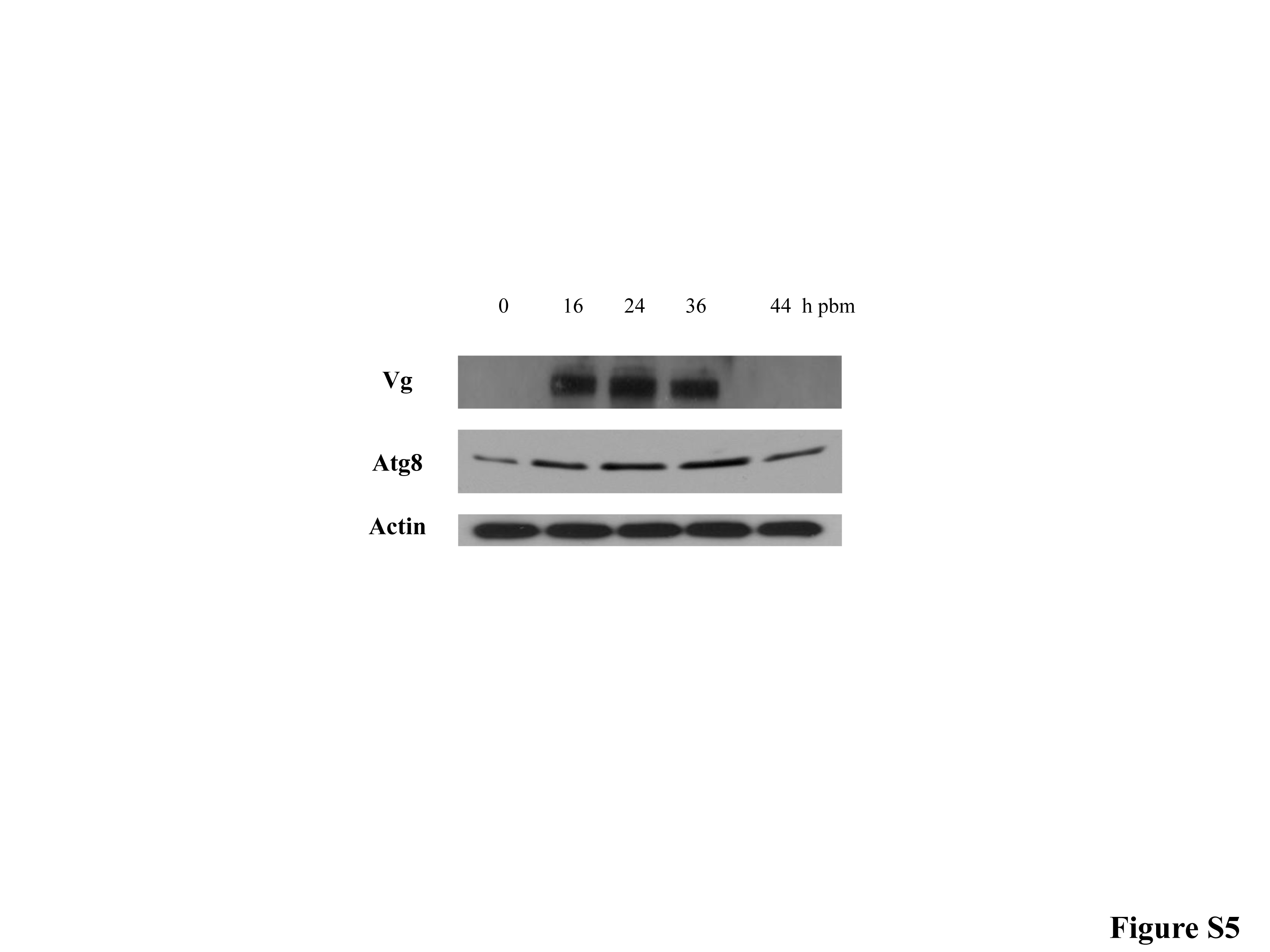

Supplement: Figure S5 — dsMal has no effect on progression of vitellogenesis. Vg, ATG8 and actin were visualized by their respective antibodies in fat bodies from mosquitoes at 0–44 h PBM. (TIF) [file pone.0025502.s005.tif]

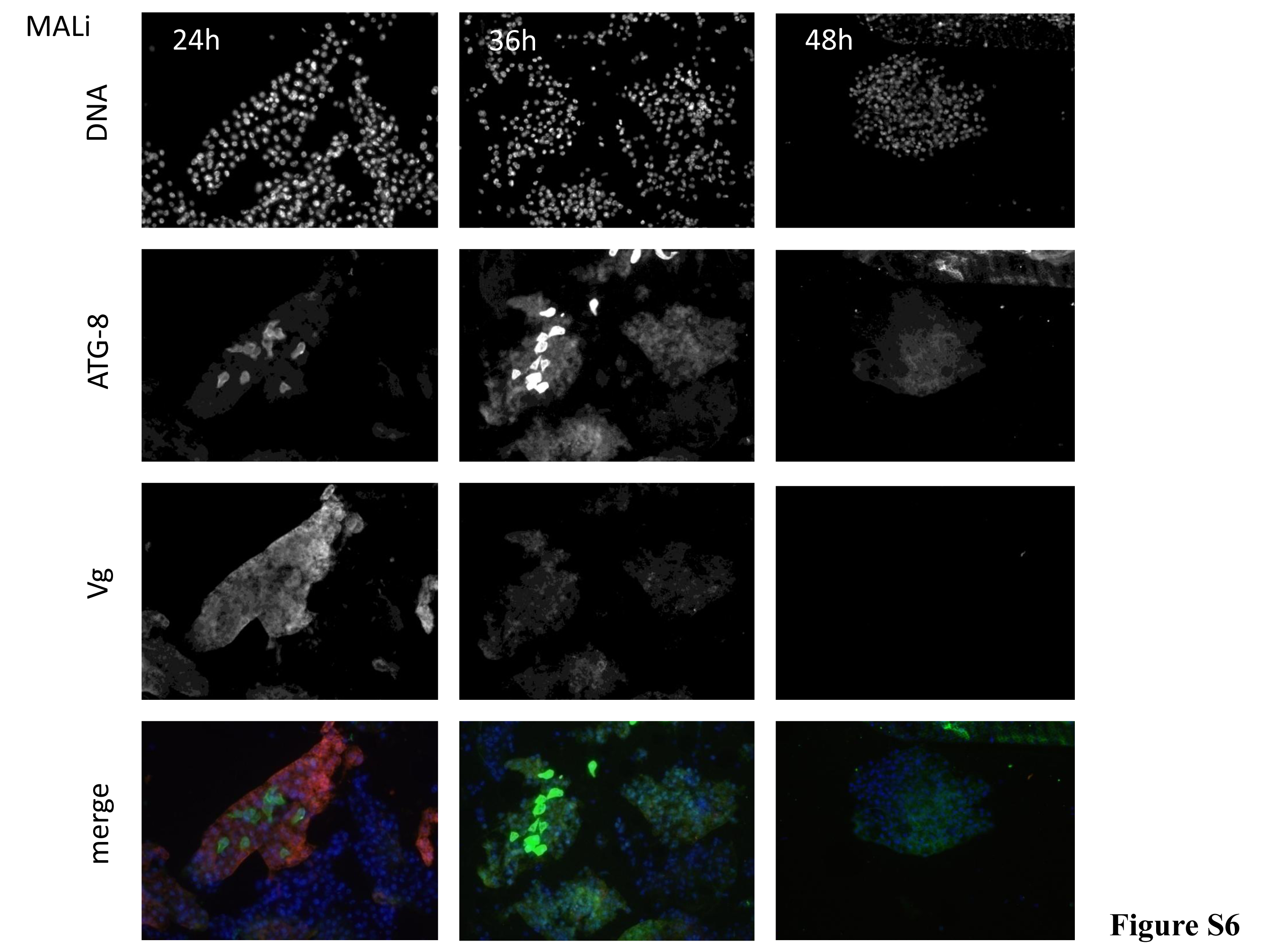

Supplement: Figure S6 — Vg and ATG8 immunofluorescence analysis in MALi background. ATG8 and Vg expression was assessed by immunofluorescence within the fat body at 24, 36 and 48 h PBM in MALi background. ATG8 was labeled with polyclonal ATG8 antibody followed by anti-rabbit FITC-conjugated antibodies (green) and Vg was labeled with Vg monoclonal antibodies followed by anti-mouse Texas-RED-conjugated antibodies (red). (TIF) [file pone.0025502.s006.tif]

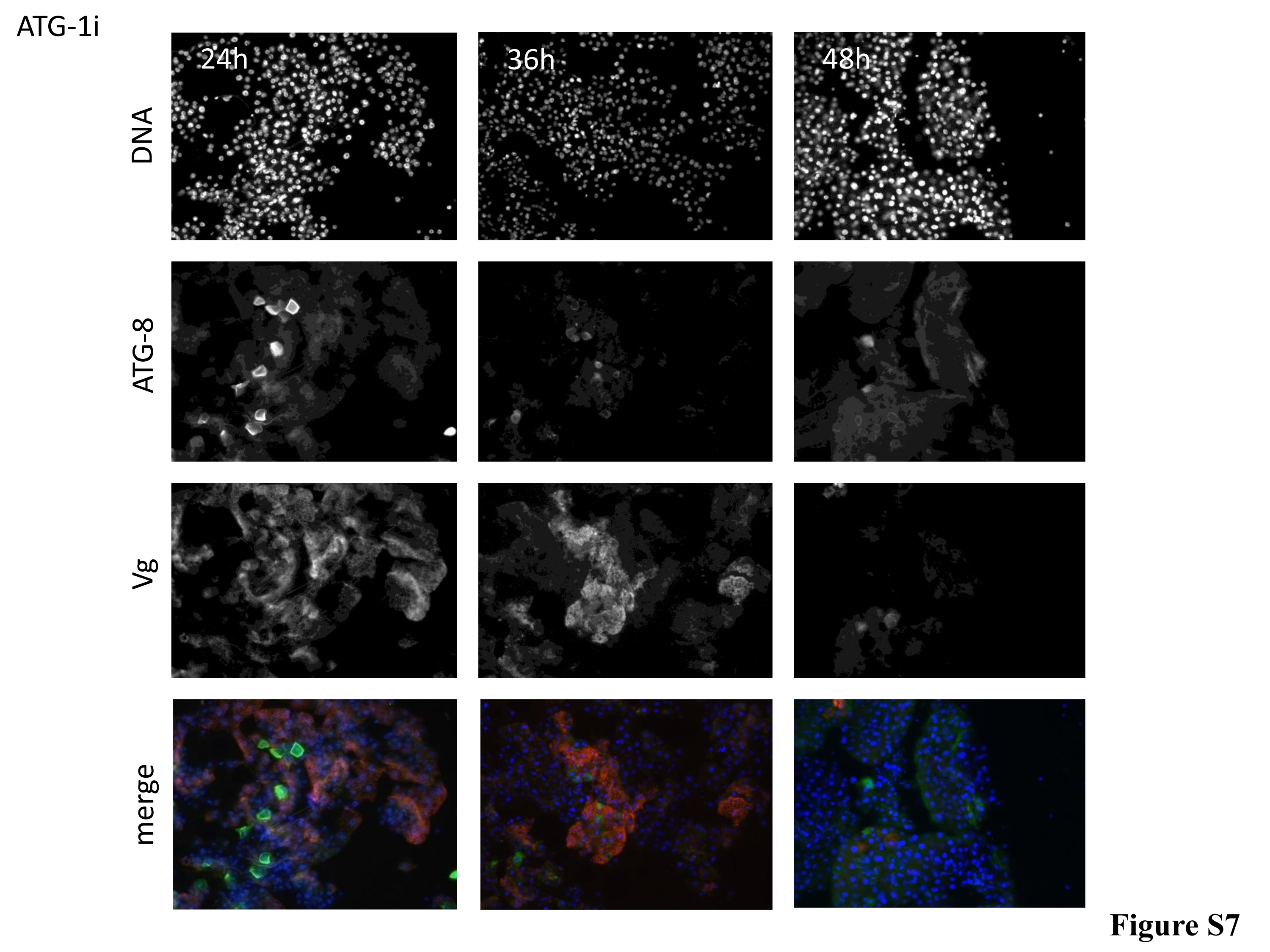

Supplement: Figure S7 — Vg and ATG8 immunofluorescence analysis in ATG1i background. ATG8 and Vg expression was assessed by immunofluorescence within the fat body at 24, 36 and 48 h PBM in ATG1i background. ATG8 was labeled with polyclonal ATG8 antibody followed by anti-rabbit FITC-conjugated antibodies (green) and Vg was labeled with Vg monoclonal antibodies followed by anti-mouse Texas-RED-conjugated antibodies (red). (TIF) [file pone.0025502.s007.tif]

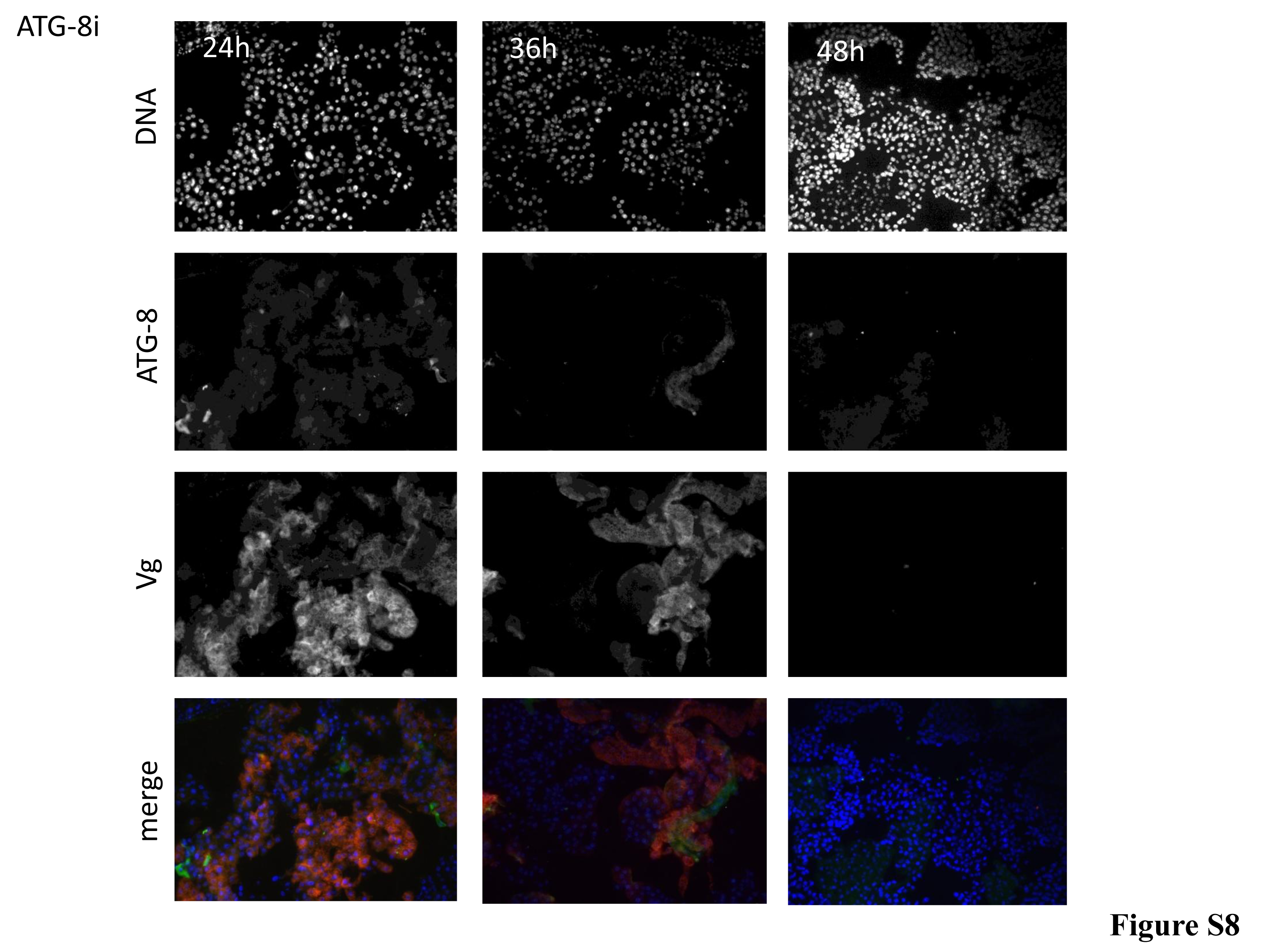

Supplement: Figure S8 — Vg and ATG8 immunofluorescence analysis in ATG8i background. ATG8 and Vg expression was assessed by immunofluorescence within the fat body at 24, 36 and 48 h PBM in ATG8i background. ATG8 was labeled with polyclonal ATG8 antibody followed by anti-rabbit FITC-conjugated antibodies (green) and Vg was labeled with Vg monoclonal antibodies followed by anti-mouse Texas-RED-conjugated antibodies (red). (TIF) [file pone.0025502.s008.tif]

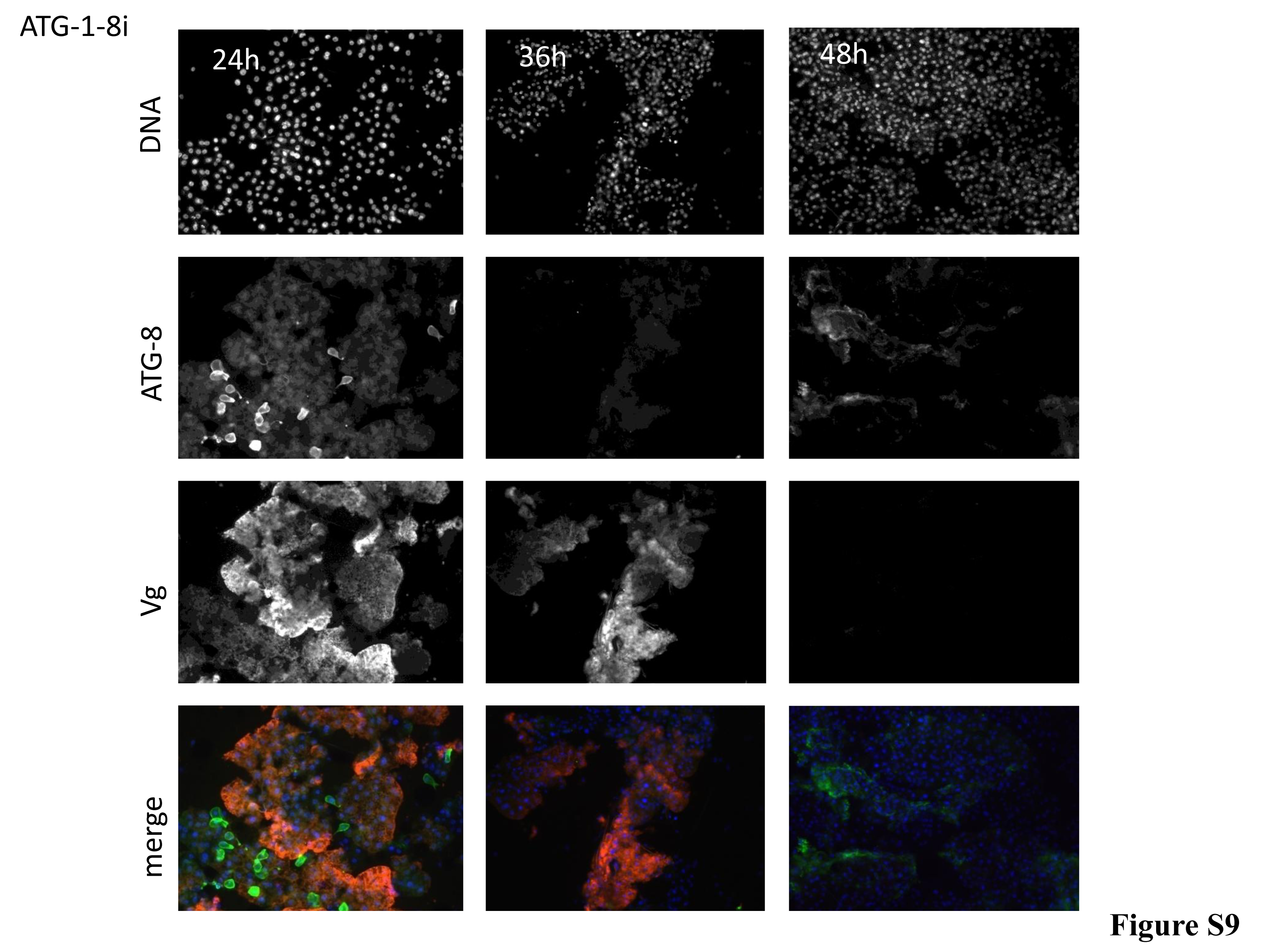

Supplement: Figure S9 — Vg and ATG8 immunofluorescence analysis in ATG1+8i background. ATG8 and Vg expression was assessed by immunofluorescence within the fat body at 24, 36 and 48 h PBM in ATG1+8i background. ATG8 was labeled with polyclonal ATG8 antibody followed by anti-rabbit FITC-conjugated antibodies (green) and Vg was labeled with Vg monoclonal antibodies followed by anti-mouse Texas-RED-conjugated antibodies (red). (TIF) [file pone.0025502.s009.tif]

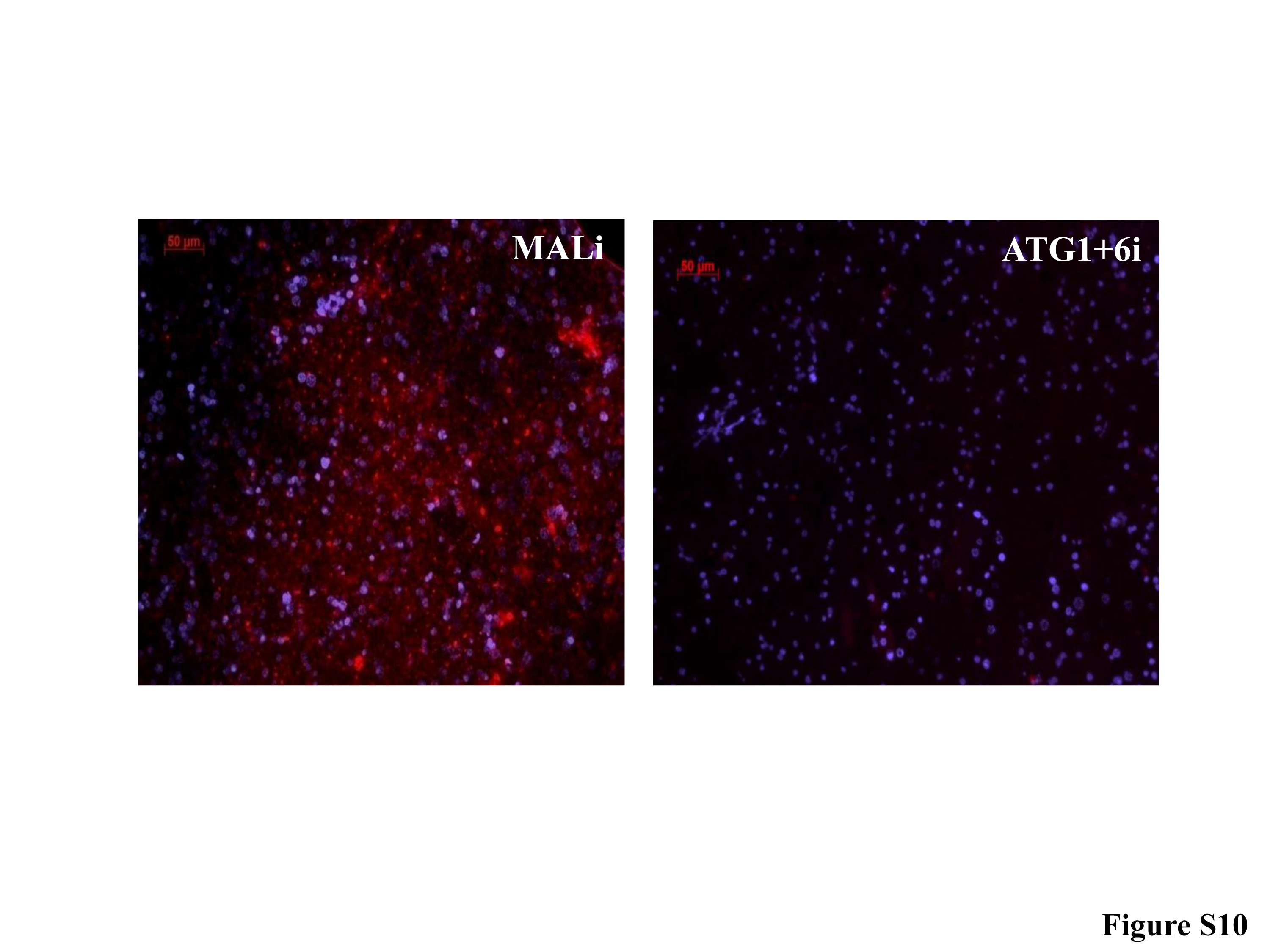

Supplement: Figure S10 — Autophagy-incompetent background ATG1+6i are unable to properly induce autophagy. Fat bodies from MALi or ATG1+6i backgrounds were assessed for lysotracker staining 36 hr PBM. Scale bar of 50 µm is shown in red. (TIF) [file pone.0025502.s010.tif]

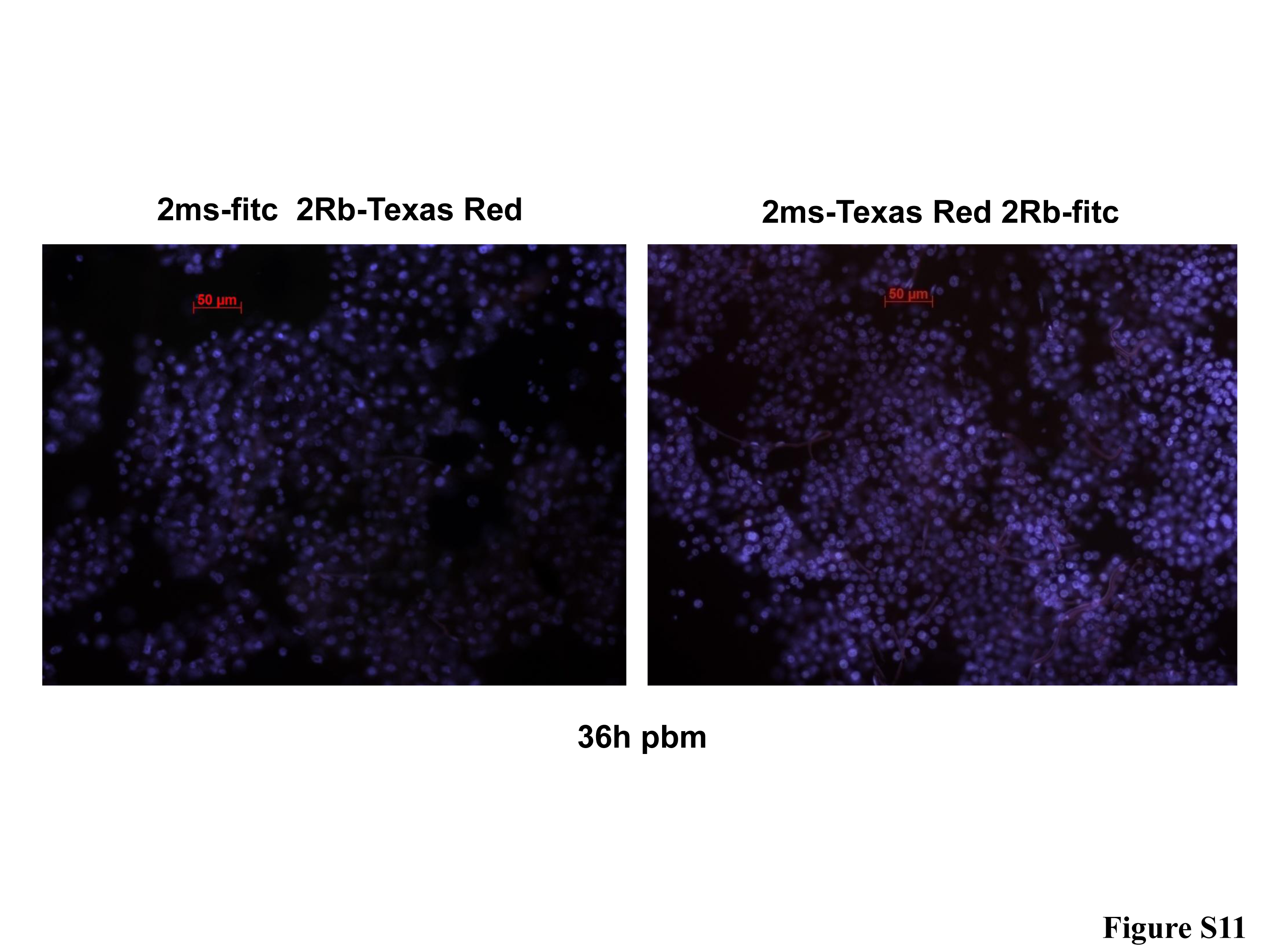

Supplement: Figure S11 — Control testing of secondary antibody staining of the fat body at 36 hr PBM in the absence of primary antibody. Fat bodies from blood fed mosquitoes at 36 h PBM were incubated with secondary antibodies without any primary antibody to illustrate the lack of non-specific binding for these antibodies. Scale bar of 50 µm is shown in red. (TIF) [file pone.0025502.s011.tif]

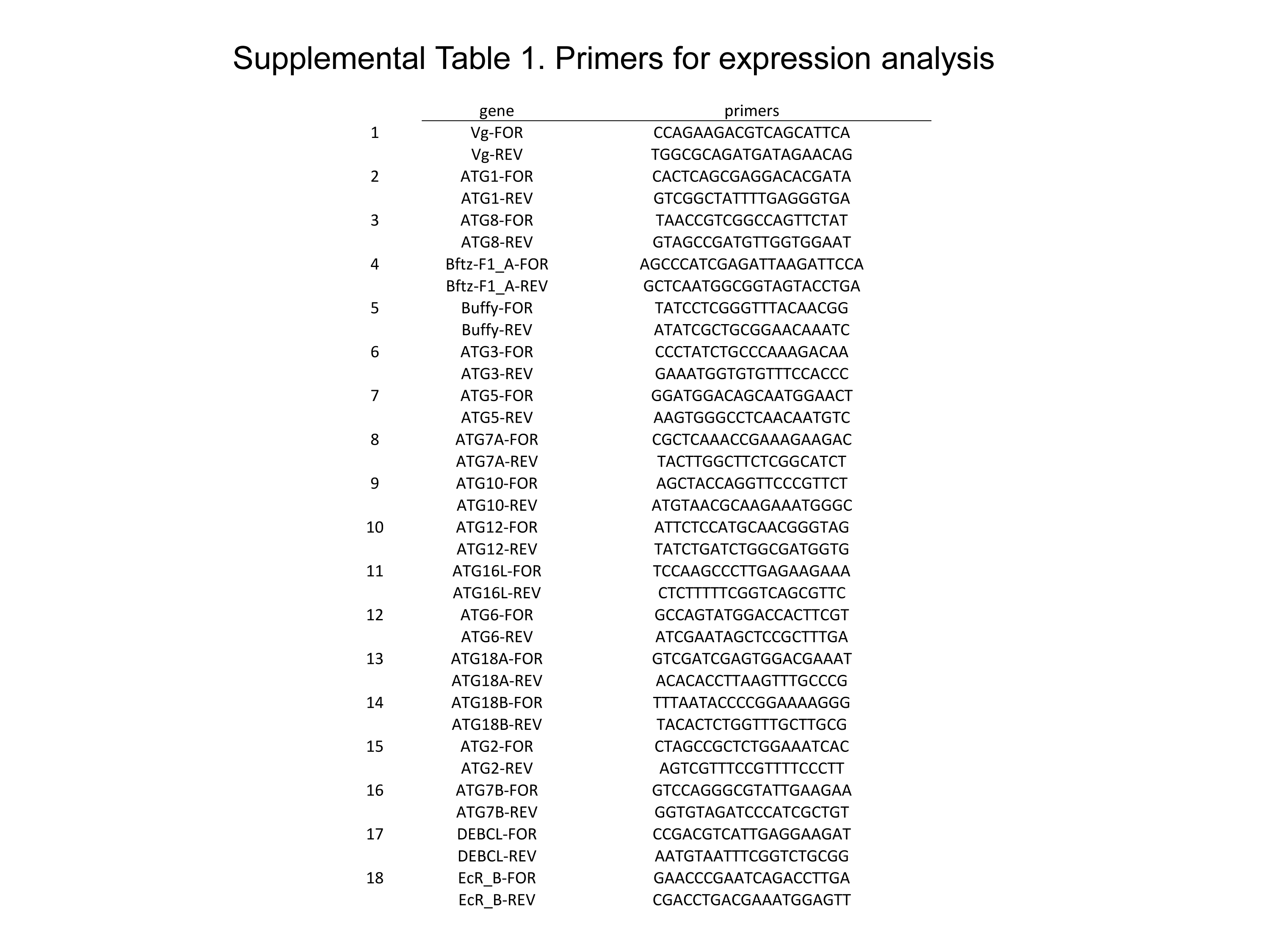

Supplement: Table S1 — Primers for expression analysis. (TIF) [file pone.0025502.s012.tif]
